# Formulations of desensitizing toothpastes for dentin hypersensitivity: a scoping review^*^

**DOI:** 10.1590/1678-7757-2021-0410

**Published:** 2022-02-07

**Authors:** Carolina Castro Martins, John Joseph Riva, Ramon Targino Firmino, Holger Jens Schünemann

**Affiliations:** 1 Universidade Federal de Minas Gerais Belo Horizonte Brasil Universidade Federal de Minas Gerais, Belo Horizonte, Brasil.; 2 McMaster University Hamilton Canada McMaster University, Hamilton, Canada.; 3 UNIFACISA Centro Universitário Campina Grande Brasil UNIFACISA Centro Universitário, Campina Grande, Brasil.

**Keywords:** Systematic review, Network meta-analysis, Meta-analysis, Dentine sensitivity, Dentifrice

## Abstract

**Objective:**

This study aimed to review evidence from randomized controlled trials (RCTs) to describe: 1) the active ingredients and desensitizing toothpaste brands; 2) the evaluation of these active ingredients over time, and 3) the fluoride and abrasive content in the formulations designed to treat dentin hypersensitivity (DH).

**Methodology:**

In total, 138 RCTs and their tested toothpastes were included. Searches were updated up to August 19, 2021. Formulations, reported brands, active ingredients over time, and type of fluoride (ionizable or ionic fluoride) and abrasive (calcium or silica-based) were analyzed (PROSPERO #CRD42018086815).

**Results:**

Our trials assessed 368 toothpaste formulations, including 34 placebo (9%), 98 control toothpastes with fluoride (27%), and 236 (64%) with active ingredients to treat DH. We tested the following active ingredients: potassium compounds (n=68, 19%), calcium sodium phosphosilicate (CSP) (n=37, 10%), strontium compounds (n=28, 8%), arginine (n=29, 8%), stannous fluoride (SnF2) (n=21, 6%), hydroxyapatite (n=9, 2%), potassium combined with another active ingredient (n=19, 5%), inorganic salt compounds (n=11, 3%), citrate (n=5, 1%), formaldehyde (n=3, 1%), herbal (n=4, 1%), copolymer (n=1, 0.5%), and trichlorophosphate (TCP) (n=1, 0.5%). The number of toothpaste formulations increased since 1968, with the greatest increment after 2010. Most toothpastes described their type of fluoride as sodium monofluorphosphate (MFP) (n=105, 29%) and NaF (n=82, 22%), with silica-based (n=84, 23%) and calcium-based (n=64, 17%) abrasives.

**Conclusion:**

Patients and dentists enjoy an increasing number of brands and active ingredients to decide what desensitizing toothpaste to use. The most common types of fluoride are MFP and NaF.

## Introduction

Over time, toothpaste brands have added several active ingredients to their products, depending on their purpose: anti-caries, antiplaque, antigingivitis, anti-malodor, antitartar, and whitening agents.^1^ Other purposes include, among others, adding more efficacious active ingredients to decrease pain from dentin hypersensitivity (DH). Systematic reviews have compared desensitizing toothpastes to treat DH with varying results.^2-5^ A recent systematic review of 125 randomized clinical trials (RCTs), which included a network meta-analysis (NMA) of 90 RCTs, concluded that calcium sodium phosphosilicate (CSP), stannous fluoride (SnF_2_) and potassium compounds in combination with hydroxyapatite or SnF_2_ were the most effective active ingredients against tactile and air stimuli with a high to moderate certainty of evidence.^2^ CSP was also effective against cold stimuli. Arginine was effective against air stimuli, and potassium and strontium compounds, for tactile stimuli (with moderate certainty of evidence). The following active ingredients showed from large to small beneficial effects when compared to fluoride with low or very low certainty and, for this reason, were considered ineffective: herbal, hydroxyapatite, inorganic salts, copolymer, and trichlorophosphate (TCP).^2^ Over-the-counter fluoride toothpastes were the most common comparator since they serve general purposes.

Our first systematic review evaluated how industry funding influenced desensitizing toothpastes trials, finding that, though the industry funded 58% of them, the funding failed to affect the directionality of results.^6^ Our second publication focused on the effectiveness of several active ingredients via an NMA.^2^ However, dentists might ask which are the brands and active ingredients available in these formulations. To our knowledge, no scoping review has described the spectrum of formulations and brands of desensitizing toothpastes. Thus, this study aimed to describe the toothpaste formulations reported by 125 included trials. Moreover, we aimed to describe: 1) active ingredients and toothpaste brands; 2) active ingredients evaluated over the years, and 3) the types of fluoride and abrasives in their formulations.

## Methodology

### Protocol and registration

This study is reported according to PRISMA for scoping reviews,^7^ and it is part of a larger systematic review registered under the protocol number PROSPERO #CRD42018086815, published elsewhere.^2^ This analysis was not reported by the original protocol and was planned later. Its protocol update was published on the PROSPERO database on August 24, 2021. We aimed to assess P (population): desensitizing toothpastes designed for patients with dentin hypersensitivity; Co (concept): the treatment of pain caused by tactile, air, and/or cold stimuli due to dentin hypersensitivity; and Co (context): assessed by RCTs.

### Eligibility criteria and information sources

RCTs evaluating the home use of desensitizing toothpastes in adult patients with DH were included in this study, in which a dentist made the clinical diagnosis of DH via tactile, cold or air stimuli. Patients who used whitening treatments or toothpastes, other non-toothpaste treatments (e.g., in office, gels, mouthwashes or laser), and those with aggressive periodontal disease or recent periodontal surgery were excluded from our review.

The electronic search from the previous review, was conducted since its inception to February 2019, and updated on August 19, 2021 for the following electronic databases: Medline, Embase, Cochrane Central Register of Controlled Trials (CENTRAL), and Cochrane Reviews. Ongoing trials in the Clinical Trials database (http://clinicaltrials.gov) and the WHO International Clinical Trials Registry Platform (ICTRP) were searched. Only ongoing studies with published data available were considered in updating this review on Clinical Trials and ICRTIP. Both ProQuest Dissertation & Theses A&I were searched for grey literature, as were the references of potentially eligible studies and previously published systematic reviews. The following keywords were combined: (dentin sensitivity OR dentin hypersensitivity OR tooth sensitivity OR teeth sensitivity) AND (dentifrice^*^ OR toothpaste^*^ OR potassium OR dentin desensitizing agent^*^ OR strontium OR arginine OR calcium OR phosphate^*^ OR sodium fluoride OR polyethylenes OR diphosphates OR calcium carbonate^*^ OR potassium nitrate OR calcium sodium phosphosilicate OR pyrophosphate^*^) AND (randomized controlled trial* OR controlled clinical trial^*^ OR random^*^ OR randomized OR placebo OR drug therapy OR randomly OR trial OR groups or meta-analysis OR systematic review^*^) NOT (animals NOT humans). The detailed search strategies for each database are published in the supplementary material of our prior systematic review (https://journals.sagepub.com/doi/suppl/10.1177/0022034520903036).^2^

### Selection of sources of evidence

Titles, abstracts, full texts, and extracted data were screened by pairs of independent reviewers. First, titles and abstracts were filtered for eligibility. Then, full texts were obtained and screened by independent reviewers. For each phase, reviewers underwent rounds of pilot calibration and training exercises led by the principal investigator (CCM). In each phase, disagreements were resolved by discussion to a consensus.

An Excel form, previously created and assessed from our prior reviews, was used to extract data, regarding: year of publication, number of trials, number of toothpastes evaluated, type of active ingredient, concentration, brands, and type of fluoride and abrasive.

### Synthesis of results

Data were transferred to the IBM SPSS software (Corp. Released 2011. IBM SPSS Statistics for Windows, Version 20.0. Armonk, NY: IBM Corp.) for statistical analysis. A descriptive analysis was conducted to estimate the frequency of each type of active ingredients. Active ingredients, and the corresponding brands reported by the trials, were ranked according to their effectiveness, as per our previous systematic review^2^. To describe the types of active ingredients evaluated over time, bar graphs which distribute them over the decades were plotted. Decades were categorized as follows: prior to 1979, 1980-1989, 1990-1999, 2000-2009, and from 2010 onwards. Lastly, the type of fluoride and abrasive was descriptively analyzed. Fluorides were categorized into: 1) ionizable: sodium monofluorphosphate (MFP), 2) ionic fluoride: sodium fluoride (NaF), and 3) fluoride-stannous complex (SnF_2_). Ionizable fluoride can usually be combined with calcium-based or silica-based formulations; and ionic fluoride can be combined with silica-based abrasives, but not with calcium-based formulations which would prevent the inactivation of the fluoride.^8^

## Results

**Figure 1:**
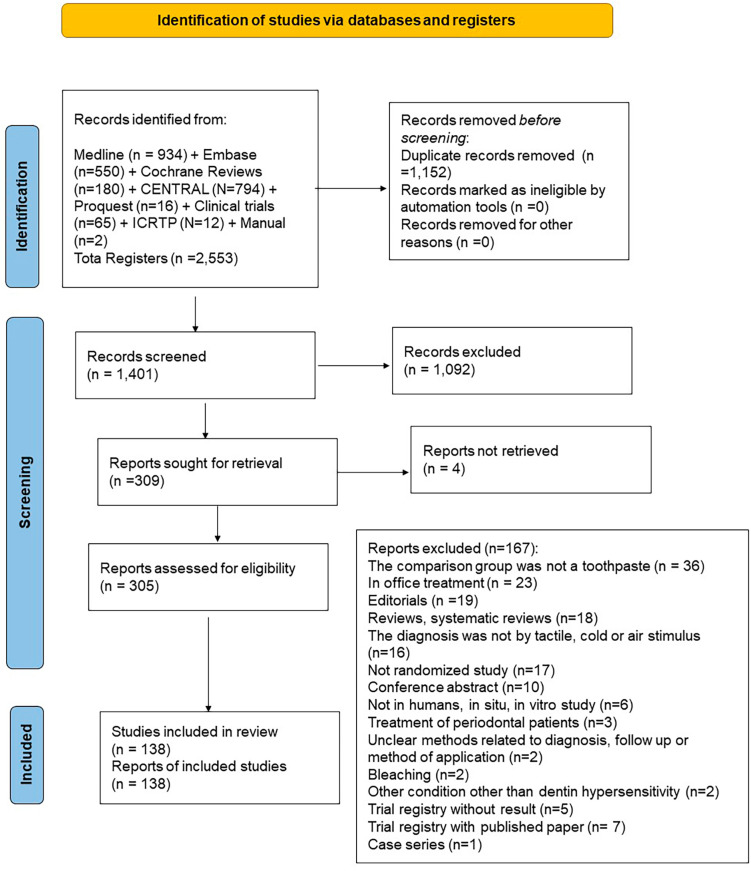
PRISMA 2020 flow diagram showing our screening process

**Figure 2:**
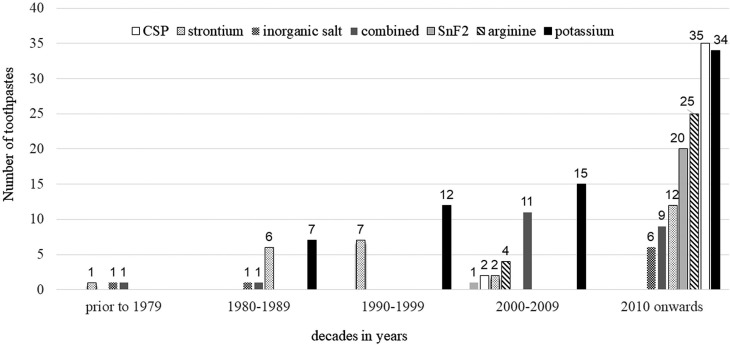
Distribution of the most common active ingredients studied over the decades. CSP: calcium sodium phosphosilicate; SnF2: stannous fluoride; Combined: potassium compounds + copolymer, potassium compounds + herbal, potassium compounds + hydroxyapatite, potassium compounds + inorganic salt, potassium compounds + SnF2, potassium compounds + strontium compounds, and inorganic salt + SnF2

Our previous review included 125 RCTs. After the current update, this scoping review includes 138 RCTs. Appendix 1 shows the list of included studies. Figure 1 shows our screening process and Appendix 2, the list of studies excluded and the reasons for their exclusion. Trials included 368 toothpastes assessed between 1968 and 2021 (summing treatment and control toothpastes): 236 (64%) had active ingredients against DH, 98 (27%) were fluoride toothpastes used as control, and 34 (9%) were placebo toothpastes (with abrasives but without fluoride or any active ingredient). Figure 2 shows the most common active ingredients: potassium compounds (n=68, 19%), CSP (n=37, 10%), strontium compounds (n=28, 8%), arginine (n=29, 8%), and SnF_2_ (n=21, 6%). In total, 22 (6%) combined two active ingredients: either potassium compounds with SnF_2_, copolymer, herbal, hydroxyapatite, inorganic salts or strontium compounds or SnF_2_ with inorganic salts.

We analyzed trials conducted between 1968 and 2021 (Figure 2), and found that the number of desensitizing toothpastes trials evaluated had increased over the decades (considering a total of 236 desensitizing toothpastes (100%)): 3 (1%) in 1968-1979, 15 (6%) in 1980-1989, 19 (8%) in the 1990-1999, 35 (15%) in 2000-2009, and 140 (60%) after 2010. Trials found other active ingredients in lower proportions (not shown in the graph): hydroxyapatite (n=9, 4%), citrate (n=5, 2%), herbal (n=4, 1%), formaldehyde (n=3, 1%) copolymer (n=1, 1%), and trichlorophosphate (TCP) (n=1, 1%). Since the 1960s, the use of potassium and strontium compounds increased over time. Arginine, CSP, and SnF_2_ are new active ingredients that appeared combined with other active ingredients after 2000.

**Figure 3:**
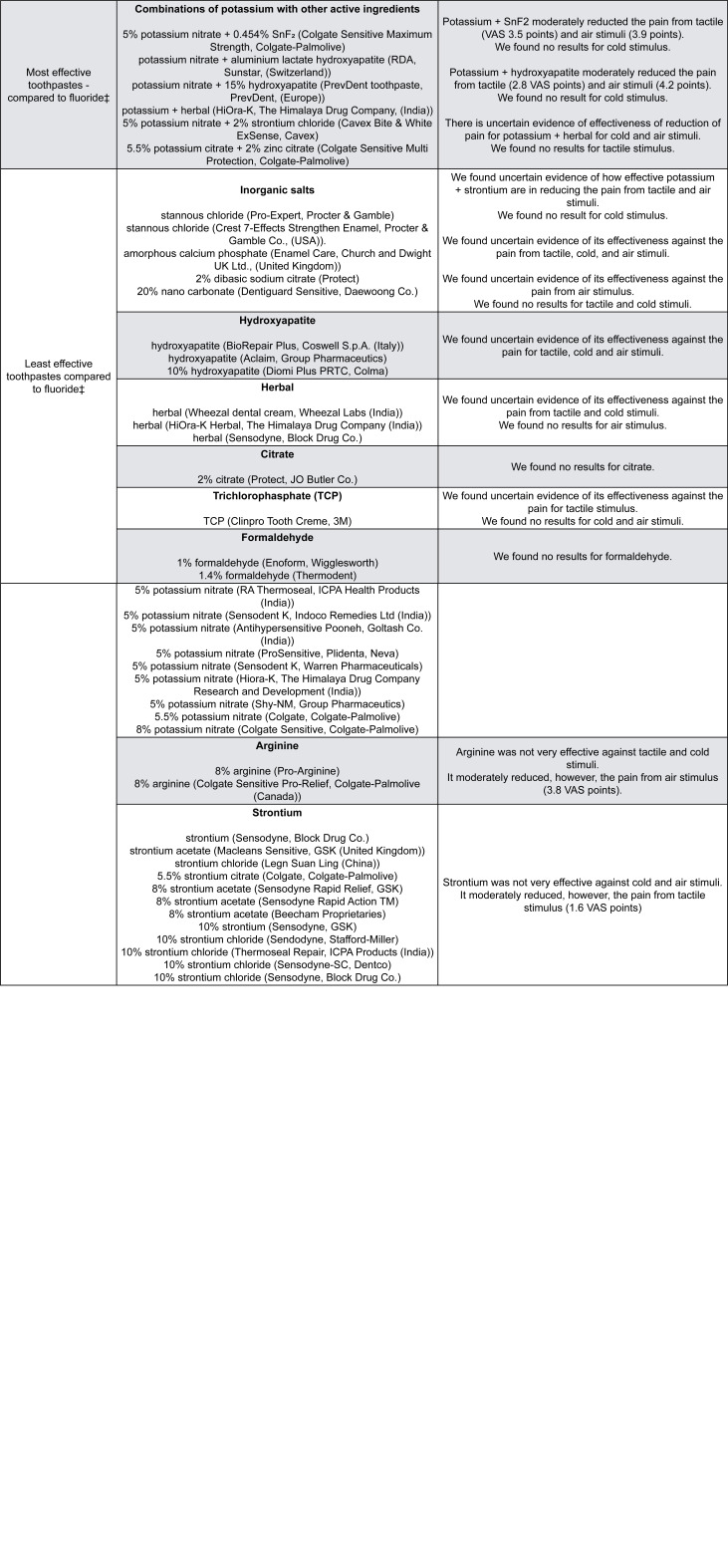
Description of the formulations of desensitizing toothpastes according to their effectiveness reported by RCTs

Figure 3 orders the reported active ingredients and brands by their effectiveness. Not all trials reported all the relevant information, such as concentration of active ingredients or all manufacturer’s details. Figure 3 clinically interprets the reduction in pain compared to standard fluoride toothpastes.^2^ We use a Visual Analogue Scale (VAS) to show the pain patients reported (varying from 0 (no pain) to 10 cm (maximum pain)). For example, the most effective toothpaste (CSP) reduces the pain air stimuli cause by 3.4 VAS points ,^2^ and probably reduces the pain of tactile and cold stimuli by 2.5 and 4.4 points, respectively. If we consider these values in percentages, the reduction in pain may vary from 25% (tactile) to 34% (air) or 44% (cold stimulus).

* Not all RCTs reported all information (e.g. concentration, manufacturer). ‡ Toothpastes were ordered by effectiveness based on the previous systematic review.2 Pain was measured by Visual Analogue Scale (VAS) that varies from 0 (no pain) to 10 cm (maximum pain) and was reported by patients before and after the follow up treatment. The evidence reported in this table was published in the supplementary material of Martins, et al.^2^ (2020) and it is based on the results of the network meta-analysis. The evidence takes into account the type of active ingredient independent of the brand.

Out of 368 toothpastes, the literature reported the following types of fluoride: MFP (n=105, 29%), NaF (n=82, 22%), SnF_2_ (n=27, 7%), NaF combined with SnF_2_ (n=5, 1%), and amine fluoride (n=3, 1%). In total, 112 (30%) trials had no such information. The literature also report the following types of abrasives: silica-based (n=84, 23%), calcium-based (n=64, 17%), diatomaceous earth (n=3, 0.8%), titanium dioxide (n=1, 0.3%), sodium triphosphate (n=1, 0.3%), and titanium dioxide combined with silica (n=2, 0.5%). In total, 213 (58%) trials lacked information on their type of abrasive. Only 115 (31%) toothpastes reported a type of fluoride combined with a type of abrasive. Among these, we found ionizable fluoride combined with calcium-based (n=52, 81%) or silica-based formulations (n=12, 19%), and ionic fluoride combined with silica-based formulations (n=48, 94%) and SnF_2_ with calcium-based formulations (n=3, 6%).

## Discussion

Over the years, numerous RCTs have evaluated the many formulations for desensitizing toothpastes. As far as we know, this is the first scoping review describing the spectrum of desensitizing toothpaste formulations assessed by trials over the years. In the absence of studies with similar methodology and results, we compared our results with studies describing the history of isolated active ingredients.

CSP and arginine were the most common toothpastes evaluated in recent years. The most common active ingredients found in this review show two basic active mechanisms for desensitizing toothpastes: arginine, CSP, and SnF_2_ occlude the dentin tubules, avoiding the propagation of fluids into them. Potassium acts by diminishing the excitability of pulpal nerves, decreasing pain sensation.^9^ Strontium has a dual function, it can both precipitate insoluble metals on dentin tubules and depolarize dental nerves.^10^ SnF_2_ can also have additional effects, such as antiplaque, antigingivitis, antimicrobial, and anticaries ones.^11,12^ Although SnF_2_ can stain teeth due to the improper stabilization of stannous ions (Sn^+^), an *in vitro* study reports that adding zinc phosphate can stabilize the formulation and reduce staining.^11^ Its desensitizing effect stems from the deposition of a chemical insoluble layer of stannous salts under topical applications.^13^

CSP and arginine are the active ingredients in most commonly assessed in recent years. We found trials testing CSP after the year 2000, which was first invented at the University of Florida in the late 1960s and is known as NovaMin Technology.^14^ CSP was created to ensure a Ca:P molar ratio similar to hydroxyapatite in bone mineral.^14^ CSP was the most effective desensitizing toothpaste in relieving pain due to tactile, cold, and air stimuli in our previous NMA.^2^ The biological rationale is that it acts as a bioactive glass reacting with salivary Ca^+^ and HPO_4_^-2^ ions, forming an amorphous layer of Ca-P, the most similar structure to enamel hydroxyapatite.^15^ Trials also began testing arginine after the year 2000. Arginine is effective in reducing air stimuli,^2^ and can transport calcium and phosphate into dentin tubules, forming a protective salivary glycoprotein with calcium and phosphate.^10^ Trials have tested much fewer formulations with both these new active ingredients, such as CSP and arginine, than those with potassium and strontium compounds, among the oldest active ingredients in use and with more types, brands, and concentrations available. Strontium compounds were one of the first active ingredients to be introduced in the early 1900s, known to strengthen teeth and to reduce sensitivity,^1^ at a time lacking strong available scientific evidence. Trials still study both these active ingredients.

So far, we lack a clear rationale to why some toothpastes are more effective for one stimulus over others, but we can consider a few of them. Each patient may respond differently to different stimuli. Also, air stimuli are the most common outcome used in clinical trials, as it is easily used in dental offices via triple syringes.^16^ As confirmed in our previous NMA, trials most often measured air stimuli (85 trials for air stimuli compared to 71 for tactile stimuli and 16 for cold stimuli).^2^ Moreover, air stimuli can affect a larger area of the dentin and thereby cause more pain.^16^ Conversely, cold stimuli are the most common pain trigger for patients,^10^ though they may be more difficult to measure in clinical trials. Tactile stimuli often relies on the use of a Yeaple or Jay probe to apply increasing force to an exposed area of the tooth^17^ or a constant manual force to probe the dental surface.^2^ We postulate that the first probe method is more accurate than the second, which depends upon the operator’s manual pressure. Overall, dentin hypersensitivity is difficult to objectively measure and evaluate since reports of pain are subjective and clinical responses to the stimuli measured in diverse ways could lead to heterogeneity.^18^

As expected, all toothpastes had fluoride (except the placebo) since it is important to prevent caries.^19^ In general, NaF or SnF_2_ were combined with silica-based formulations and we found some SnF_2_ toothpastes combined with calcium-based formulations. If ionic fluoride (e.g., NaF) is combined to calcium-based formulations, the free F^-^ ion reacts with Ca^+2^ forming calcium fluoride (CaF_2_) which is a insoluble fluoride for caries prevention. Also, calcium-based formulations are combined with ionizable fluoride (MFP), although fluoride can hydrolyze and release free F^-^ to form inactive CaF_2_ over time.^8,20^ However, we observed no improper combinations of fluoride with abrasives in the sample of toothpastes analyzed. Based on this information and on our results, we assume that these toothpastes are stable and effective in also preventing dental caries.

We previously described that the most effective toothpastes, compared to fluoride toothpastes (moderate to high certainty of evidence), were: CSP, SnF_2_, and potassium compounds combined with SnF_2_ or hydroxyapatite.^2^ We found few options for the combined active ingredients, possibly because the trials were testing future combinations. Dental companies funded 58% of trials but industry funding was unrelated to positive results.^6^ Many factors likely influence how consumers make choices for or against a toothpaste brand. One study reported that patients choose toothpastes mainly due to the brand name and to their dentists’ advice.^21^ Advertisement is likely to play a role as well.

### Strengths and limitations

This is the first review that used a scoping methodology to describe the active ingredients, formulations, and brands of desensitizing toothpastes. We found no other study in the literature describing the spectrum of desensitizing toothpaste formulations. This study lacks a chemical lab test to check the reported label formulations. However, as this is a scoping review, we had to rely on authors’ information. Also, a high percentage of information was missing, such as concentration values and brand names. Moreover, 30% (n=112) of toothpastes lacked information on the specific type of fluoride used and 58% (n=213) on the particular type of the abrasive used. Only 32% (n=115) of toothpastes fully reported this information. Due to the missing information, we were unable to perform a more rigorous statistical analysis of the combination of fluorides and abrasives, and to categorize toothpastes according to concentration values. In the future, we strongly suggest that trials report complete formulation breakdowns.

### Implications for research and clinical practice

For future research, trials should report the full formulation (active ingredients and concentrations) of fluorides and abrasives. A recent NMA showed that combinations of two active ingredients, such as potassium compounds with hydroxyapatite or SnF_2_, were efficient against DH, though it included too few trials to base this evidence.^2^ Thus, more studies testing the combination of the active ingredients of toothpastes may further clarify their effectiveness. This review provides clinical dentists with a list of the most effective desensitizing toothpastes together with the best available evidence for patients and their dentists to make decisions together. The decision of which toothpaste to use should consider the type of pain patients report, the scientific evidence, the toothpastes available in their jurisdiction and the long-term cost for patients. We acknowledge that desensitizing toothpastes are more expensive than traditional fluoride ones. However, these products are still less expensive than in-office treatments for dentin hypersensitivity and the reported side effects of toothbrushing with them are very rare.^2^ In summary, toothbrushing with desensitizing toothpastes offers more benefits than harms for patients with dentin hypersensitivity.

## Conclusion

There are increasingly more brands of desensitizing toothpastes on the market. This study categorized the active ingredients in these formulations according to their effectiveness, and MFP and NaF were the most common types of fluoride desensitizing toothpastes.

## Appendix 1 - References of the studies included in our systematic review



## Appendix 2 - Studies excluded (with reasons) after full-text analysis


