# Supplementary material for: Formulations of desensitizing toothpastes for dentin hypersensitivity: a scoping review
Source: J Appl Oral Sci. 2022 Feb 7;30:e20210410. doi: 10.1590/1678-7757-2021-0410 (PMC8908863; doi:10.1590/1678-7757-2021-0410)
Supplement: Supplementary file 1 [file 1678-7757-jaos-30-e20210410-app01.pdf]

Systematic Review
http://dx.doi.org/10.1590/1678-7757-2021-0410

JAOS
JOURNAL OF APPLIED ORAL SCIENCE

Appendix 1 - References of the studies included in our systematic review

1 - Acharya AB, Surve SM, Thakur SL. A clinical study of the effect of calcium sodium phosphosilicate on dentin hypersensitivity. J Clin Exp Dent. 2013;5(1):e18-22. doi: 10.4317/jced.50955

2 - Addy M, Mostafa P, Newcombe R. Dentine hypersensitivity: a comparison of five toothpastes used during a 6-week treatment period. Br Dent J. 1987;163(2):45-51. doi: 10.1038/sj.bdj.4806185

3 - Al Habashneh R, Farasin R, Khader Y. The effect of a triclosan/copolymer/fluoride toothpaste on plaque formation, gingivitis, and dentin hypersensitivity: a single-blinded randomized clinical study. Quintessence Int. 2017;48(2):123-30. doi: 10.3290/j.qi.a37384

4 - Anand S, Rejula F, Sam JV, Christaline R, Nair MG, Dinakaran S. Comparative evaluation of effect of nano-hydroxyapatite and 8% arginine containing toothpastes in managing dentin hypersensitivity: double blind randomized clinical trial. Acta Medica (Hradec Kralove). 2017;60(3):114-9. doi: 10.14712/18059694.2018.3

5 - Ashwini S, Swatika K, Kamala DN. Comparative evaluation of desensitizing efficacy of dentifrice containing 5% fluoro calcium phosphosilicate versus 5% calcium sodium phosphosilicate: a randomized controlled clinical trial. Contemp Clin Dent. 2018;9(3):330-6. doi: 10.4103/ccd.ccd_735_17

6 - Athuluru D, Reddy C, Sudhir KM, Kumar K, Gomasani S, Nagarakanti S. Evaluation and comparison of efficacy of three desensitizing dentifrices on dentinal hypersensitivity and salivary biochemical characteristics: A randomized controlled trial. Dent Res J (Isfahan). 2017;14(2):150-7.

7 - Ayad F, Berta R, De Vizio W, McCool J, Petrone ME, Volpe AR. Comparative efficacy of two dentifrices containing 5% potassium nitrate on dentinal sensitivity: a twelve-week clinical study. J Clin Dent. 1994;5 Spec No:97-101.

8 - Ayad F, Ayad N, Zhang YP, DeVizio W, Cummins D, Mateo LR. Comparing the efficacy in reducing dentin hypersensitivity of a new toothpaste containing 8.0% arginine, calcium carbonate, and 1450 ppm fluoride to a commercial sensitive toothpaste containing 2% potassium ion: an eight-week clinical study on Canadian adults. J Clin Dent. 2009;20(1):10-6.

9 - Ayad F, Ayad N, Delgado E, Zhang YP, DeVizio W, Cummins D, et al. Comparing the efficacy in providing instant relief of dentin hypersensitivity of a new toothpaste containing 8.0% arginine, calcium carbonate, and 1450 ppm fluoride to a benchmark desensitizing toothpaste containing 2% potassium ion and 1450 ppm fluoride, and to a control toothpaste with 1450 ppm fluoride: a three-day clinical study in Mississauga, Canada. J Clin Dent. 2009;20(4):115-22.

10 - Bansal D, Mahajan M. Comparative evaluation of effectiveness of three desensitizing tooth pastes for relief in the dentinal hypersensitivity. Contemp Clin Dent. 2017;8(2):195-199. doi: 10.4103/ccd.ccd_135_17

11 - Bhowmik E, Pawar Chandrashekhar D, Sharma Hareesha M. Comparative evaluation of fluorinol and calcium sodium phosphosilicate-containing toothpastes in the treatment of dentin hypersensitivity. Int J Dent Hyg. 2021;19(4):421-8. doi: 10.1111/idh.12495

12 - Bolden TE, Volpe AR, King WJ. The desensitizing effect of a sodium monofluorophosphate dentifrice. Periodontics. 1968;6(3):112-4.

13 - Chaknis P, Panagakos FS, DeVizio W, Sowinski J, Petrone D, Proskin H. Assessment of hypersensitivity reduction of a dentifrice containing 0.3% triclosan, 2.0% PVM/MA copolymer, 0.243% NaF and specially-designed silica as compared to a dentifrice containing 0.454% stannous fluoride, sodium hexametaphosphate and zinc lactate and to a dentifrice containing 0.243% NaF on dentin hypersensitivity reduction: an 8-week study. Am J Dent. 2011 Jul;24 Spec No A:14A-20A.

14 - Clark DC, Al-Joburi W, Chan EC. The efficacy of a new dentifrice in treating dentin sensitivity: effects of sodium citrate and sodium fluoride as active ingredients. J Periodontal Res. 1987;22(2):89-93. doi: 10.1111/j.1600-0765.1987.tb01545.x

15 - Collins JF, Perkins L. Clinical evaluation of the effectiveness of three dentifrices in relieving dentin sensitivity. J Periodontol. 1984 Dec;55(12):720-5. doi: 10.1902/jop.1984.55.12.720

16 - Conforti N, Battista GW, Petrone DM, Petrone ME, Chaknis P, Zhang YP, wt al. Comparative investigation of the desensitizing efficacy of a new dentifrice: a 14-day clinical study. Compend Contin Educ Dent Suppl. 2000;(27):17-22; quiz 28.

17 - Creeth J, Maclure R, Seong J, Gomez-Pereira P, Budhawant C, Sufi F, et al. Three randomized studies of dentine hypersensitivity reduction after short-term SnF2 toothpaste use. J Clin Periodontol. 2019;46(11):1105-15. doi: 10.1111/jcpe.13175

18 - Creeth J, Gallob J, Sufi F, Qaqish J, Gomez-Pereira P, Budhawant C, et al. Randomised clinical studies investigating immediate and short-term efficacy of an occluding toothpaste in providing dentine hypersensitivity relief. BMC Oral Health. 2019;19(1):98. doi: 10.1186/s12903-019-0781-x

19 - Creeth JE, Burnett GR. Efficacy of an experimental occlusion technology toothpaste in the relief of dentinal hypersensitivity: an 8-week randomised controlled trial. Oral Health Prev Dent. 2021;19(1):195-202. doi: 10.3290/j.ohpd.b1075109

20 - Day T, Einwag J, Hermann JS, He T, Anastasia MK, Barker M, et al. A clinical assessment of the efficacy of a stannous-containing sodium fluoride dentifrice on dentinal hypersensitivity. J Contemp Dent Pract. 2010;11(1):E001-8.
21 - Devi Bala RT, Victor DJ, Subramaniam S, Prakash PS, Abirami T. Comparision of commercially available desensitizing toothpastes in the management of dentin hypersensitivity-a randomized controlled clinical trial. J Pharmaceut Scie Res. 2019;11(5):1787-9.

22 - Docimo R, Montesani L, Maturo P, Costacurta M, Bartolino M, DeVizio W, et al. Desensitizing efficacy of a new toothpaste containing 5.5% potassium citrate: a 4-week clinical study. Am J Dent. 2007;20(4):209-11.

23 - Docimo R, Montesani L, Maturo P, Costacurta M, Bartolino M, Zhang YP, et al. Comparing the efficacy in reducing dentin hypersensitivity of a new toothpaste containing 8.0% arginine, calcium carbonate, and 1450 ppm fluoride to a benchmark commercial desensitizing toothpaste containing 2% potassium ion: an eight-week clinical study in Rome, Italy. J Clin Dent. 2009;20(4):137-43.

24 - Docimo R, Perugia C, Bartolino M, Maturo P, Montesani L, Zhang YP, et al. Comparative evaluation of the efficacy of three commercially available toothpastes on dentin hypersensitivity reduction: an eight-week clinical study. J Clin Dent. 2011;22(4):121-7.

25 - Du Min Q, Bian Z, Jiang H, Greenspan DC, Burwell AK, Zhong J, et al. Clinical evaluation of a dentifrice containing calcium sodium phosphosilicate (novamin) for the treatment of dentin hypersensitivity. Am J Dent. 2008;21(4):210-4.

26 - Fu Y, Li X, Que K, Wang M, Hu D, Mateo LR, DeVizio W, Zhang YP. Instant dentin hypersensitivity relief of a new desensitizing dentifrice containing 8.0% arginine, a high cleaning calcium carbonate system and 1450 ppm fluoride: a 3-day clinical study in Chengdu, China. Am J Dent. 2010;23 Spec No A:20A-27A.

27 - Fu Y, Sufi F, Wang N, Young S, Feng X. An exploratory randomised study to evaluate the efficacy of an experimental occlusion-based dentifrice in the relief of dentin hypersensitivity. Oral Health Prev Dent. 2019;17(2):107-15. doi: 10.3290/j.ohpd.a42372

28 - Gallob J, Sufi F, Amini P, Siddiqi M, Mason S. A randomised exploratory clinical evaluation of dentifrices used as controls in dentinal hypersensitivity studies. J Dent. 2017;64:80-7. doi: 10.1016/j.jdent.2017.06.009

29 - Ghassemi A, Hooper W, Winston AE, Sowinski J, Bowman J, Sharma N. Effectiveness of a baking soda toothpaste delivering calcium and phosphate in reducing dentinal hypersensitivity. J Clin Dent. 2009;20(7):203-10.

30 - Gibson M, Sharif MO, Smith A, Saini P, Brunton PA. A practice-based randomised controlled trial of the efficacy of three interventions to reduce dentinal hypersensitivity. J Dent. 2013;41(8):668-74. doi: 10.1016/j.jdent.2013.06.003

31 - Gillam DG, Newman HN, Bulman JS, Davies EH. Dentifrice abrasivity and cervical dentinal hypersensitivity. Results 12 weeks following cessation of 8 weeks' supervised use. J Periodontol. 1992;63(1):7-12. doi: 10.1902/jop.1992.63.1.7

32 - Gillam DG, Newman HN, Davies EH, Bulman JS. Clinical efficacy of a low abrasive dentifrice for the relief of cervical dentinal hypersensitivity. J Clin Periodontol. 1992;19(3):197-201. doi: 10.1111/j.1600-051x.1992.tb00639.x

33 - Gillam DG, Bulman JS, Jackson RJ, Newman HN. Comparison of 2 desensitizing dentifrices with a commercially available fluoride dentifrice in alleviating cervical dentine sensitivity. J Periodontol. 1996 Aug;67(8):737-42. doi: 10.1902/jop.1996.67.8.737

34 - Gopinath NM, John J, Nagappan N, Prabhu S, Kumar ES. Evaluation of dentifrice containing nano-hydroxyapatite for dentinal hypersensitivity: a randomized controlled trial. J Int Oral Health. 2015;7(8):118-22.

35 - Hall C, Mason S, Cooke J. Exploratory randomised controlled clinical study to evaluate the comparative efficacy of two occluding toothpastes - a 5% calcium sodium phosphosilicate toothpaste and an 8% arginine/calcium carbonate toothpaste - for the longer-term relief of dentine hypersensitivity. J Dent. 2017;60:36-43. doi: 10.1016/j.jdent.2017.02.009

36 - He T, Cheng R, Biesbrock AR, Chang A, Sun L. Rapid desensitizing efficacy of a stannous-containing sodium fluoride dentifrice. J Clin Dent. 2011;22(2):40-5.

37 - He T, Barker ML, Qaqish J, Sharma N. Fast onset sensitivity relief of a 0.454% stannous fluoride dentifrice. J Clin Dent. 2011;22(2):46-50.

38 - He T, Chang J, Cheng R, Li X, Lily S, Biesbrock AR. Clinical evaluation of the fast onset and sustained sensitivity relief of a 0.454% stannous fluoride dentifrice compared to an 8.0% arginine-calcium carbonate-sodium monofluorophosphate dentifrice. Am J Dent. 2011;24(6):336-40.

39 - He T, Barker ML, Biesbrock AR, Miner M, Qaqish J, Sharma N. A clinical study to assess the effect of a stabilized stannous fluoride dentifrice on hypersensitivity relative to a marketed sodium fluoride/triclosan control. J Clin Dent. 2014;25(2):13-8.

40 - He T, Barker ML, Biesbrock A, Sharma N. A randomized controlled clinical trial to assess the desensitizing effect of a stannous fluoride dentifrice. Am J Dent. 2014;27(2):106-10.

41 - Hegde S, Rao BH, Kakar RC, Kakar A. A comparison of dentifrices for clinical relief from dentin hypersensitivity using the Jay Sensitivity Sensor Probe. Am J Dent. 2013;26(Spec No B):29B-36B.

42 - Hernandez F, Mohammed C, Shannon I, Volpe A, King W. Clinical study evaluating the desensitizing effect and duration of two commercially available dentifrices. J Periodontol. 1972;43(6):367-72. doi: 10.1902/jop.1972.43.6.367

43 - Hu D, Zhang YP, Chaknis P, Petrone ME, Volpe AR, DeVizio W. Comparative investigation of the desensitizing efficacy of a new dentifrice containing 5.5% potassium citrate: an eight-week clinical study. J Clin Dent. 2004;15(1):6-10.

44 - Hughes N, Mason S, Jeffery P, Welton H, Tobin M, O'Shea C, et al. A comparative clinical study investigating the efficacy of a test dentifrice containing 8% strontium acetate and 1040 ppm sodium fluoride versus a marketed control dentifrice containing 8% arginine, calcium carbonate, and 1450 ppm sodium monofluorophosphate in reducing dentinal hypersensitivity. J Clin Dent. 2010;21(2):49-55.

45 - Kakar A, Kakar K, Sreenivasan P, DeVizio W, Kohli R. Comparison of the clinical efficacy in reducing dentin hypersensitivity of a new dentifrice containing 8.0% arginine, calcium carbonate, and 1000 ppm sodium monofluorophosphate to a commercially available toothpaste containing 1000 ppm sodium monofluorophosphate: an eight-week clinical trial on adults in New Delhi, India. J Clin Dent. 2012;23(2):33-9.

46 - Kakar A, Kakar K, Sreenivasan P, DeVizio W, Kohli R. Comparison of the clinical efficacy of a new dentifrice containing 8.0% arginine, calcium carbonate, and 1000 ppm fluoride to a commercially available sensitive toothpaste containing 2% potassium ion on dentin hypersensitivity: a randomized clinical trial. J Clin Dent. 2012; 23(2):40-7.

47 - Kakar A, Dl BS, Kakar K. Clinical assessment of a new dentifrice with 8% arginine and calcium carbonate on dentin hypersensitivity in an Indian population using a new measuring device: the Jay Sensitivity Sensor Probe. Am J Dent. 2013;26(Spec issue B):13B-20B.

48 - Kakar A, Kakar K. Measurement of dentin hypersensitivity with the Jay Sensitivity Sensor Probe and the Yeaple probe to compare relief from dentin hypersensitivity by dentifrices. Am J Dent. 2013;26(Spec No B):21B-28B.

49 - Katanec T, Majstorovic M, Negovetic Vranic D, Ivic Kardum M, Marks LA. New toothpaste to deal with dentine hypersensitivity: double-blind randomized controlled clinical trial. Int J Dent Hyg. 2018;16(1):78-84. doi: 10.1111/idh.12231

50 - Kim S, Park J, Lee C, Koo K, Kim T, Seol Y, et al. The clinical effects of a hydroxyapatite containing toothpaste for dentine hypersensitivity. J Korean Acad Periodontol. 2009;39(1):87-94. doi: 10.5051/jkape.2009.39.1.87

51 - Krause C, Lunau N, Sufi F. An exploratory clinical study to monitor clinical efficacy of an occluding technology dentifrice in providing short term relief from dentinal hypersensitivity. J Clin Dent. 2018;29(1):18-22.

52 - Kumari M, Naik S, Rao N, Martande S, Pradeep A. Clinical efficacy of a herbal dentifrice on dentinal hypersensitivity: a randomized controlled clinical trial. Aust Dent J. 2013;58(4):483-90. doi: 10.1111/adj.12109

53 - Kumari M, Naik SB, Martande SS, Pradeep AR, Singh P. Comparative efficacy of a herbal and a non-herbal dentifrice on dentinal hypersensitivity: a randomized, controlled clinical trial. J Investig Clin Dent. 2016;7(1):46-52. doi: 10.1111/jicd.12133

54 - Lee S, Jung H, Jung B, Cho Y, Kwon H, Kim B. Desensitizing efficacy of nano-carbonate apatite dentifrice and Er,Cr: YSGG laser: a randomized clinical trial. Photomed Laser Surg. 2015;33(1):9-14. doi: 10.1089/pho.2014.3787

55 - Li Y, Lee S, Zhang YP, Delgado E, DeVizio W, Mateo LR. Comparison of clinical efficacy of three toothpastes in reducing dentin hypersensitivity. J Clin Dent. 2011;22(4):113-20.

56 - Li CX, Huang JY, Liang HY. Toothpaste containing NovaMin is applied to treat dentine hypersensitivity. Chin J Tissue Eng Res. 2013;17(38):6846-52. doi: 10.3969/j.issn.2095-4344.2013.38.022

57 - Liang Y, Rong WS, Wang WJ, Ge LH. [A clinical study on the effectiveness of desensitizing toothpaste in patients with dentine hypersensitivity]. J Peking Univ Health sci. 2011;43(1):112-6. Chinese.

58 - Litkowski L, Greenspan DC. A clinical study of the effect of calcium sodium phosphosilicate on dentin hypersensitivity: proof of principle. J Clin Dent. 2010;21(3):77-81.

59 - Liu H, Hu D. Efficacy of a commercial dentifrice containing 2% strontium chloride and 5% potassium nitrate for dentin hypersensitivity: a 3-day clinical study in adults in China. Clin Ther. 2012;34(3):614-22. doi: 10.1016/j.clinthera.2012.01.027

60 - Madhu P, Setty S, Ravindra S. Dentinal hypersensitivity? Can this agent be the solution? Indian J Dent Res. 2006;17(4):178-84. doi: 10.4103/0970-9290.29867

61 - Majji P, Murthy KR. Clinical efficacy of four interventions in the reduction of dentinal hypersensitivity: a 2-month study. Indian J Dent Res. 2016;27(5):477-82. doi: 10.4103/0970-9290.195618

62 - Manochehr-Pour M, Bhat M, Bissada N. Clinical evaluation of two potassium nitrate toothpastes for the treatment of dental hypersensitivity. Periodontal Case Rep. 1984;6(1):25-30.

63 - Mason S, Hughes N, Sufi F, Bannon L, Maggio B, North M, et al. A comparative clinical study investigating the efficacy of a dentifrice containing 8% strontium acetate and 1040 ppm fluoride in a silica base and a control dentifrice containing 1450 ppm fluoride in a silica base to provide immediate relief of dentin hypersensitivity. J Clin Dent. 2010;21(2):42-8.

64 - McFall WT Jr, Hamrick SW. Clinical effectiveness of a dentifrice containing fluoride and a citrate buffer system for treatment of dentinal sensitivity. J Periodontol. 1987;58(10):701-5. doi: 10.1902/jop.1987.58.10.701

65 - Minkoff S, Axelrod S. Efficacy of strontium chloride in dental hypersensitivity. J Periodontol. 1987; 58(7):470-4. doi: 10.1902/jop.1987.58.7.470.

66 - Moslemi N, Johari M, Akhoundi MS, Zare H, Shamshiri AR, Khorshidian A. Comparison of desensitizing efficacy of an Iranian dentifrice and a commercially available dentifrice: a randomized double-blinded controlled clinical trial. J Dent. 2013;10(4):351-7.

67 - Nagata T, Ishida H, Shinohara H, Nishikawa S, Kasahara S, Wakano Y, et al. Clinical evaluation of a potassium nitrate dentifrice for the treatment of dentinal hypersensitivity. J Clin Periodontol. 1994;21(3):217-21. doi: 10.1111/j.1600-051x.1994.tb00307.x

68 - Naoum SJ, Lenard A, Martin FE, Ellakwa A. Enhancing fluoride mediated dentine sensitivity relief through functionalised tricalcium phosphate activity. Int Sch Res Notices. 2015;2015:905019. doi: 10.1155/2015/905019

69 - Narongdej T, Sakoolnamarka R, Boonroung T. The effectiveness of a calcium sodium phosphosilicate desensitizer in reducing cervical dentin hypersensitivity: a pilot study. J Am Dent Assoc. 2010;141(8):995-9. doi: 10.14219/jada.archive.2010.0313

70 - Nathoo S, Delgado E, Zhang Y, DeVizio W, Cummins D, Mateo L. Comparing the efficacy in providing instant relief of dentin hypersensitivity of a new toothpaste containing 8.0% arginine, calcium carbonate, and 1450 ppm fluoride relative to a benchmark desensitizing toothpaste containing 2% potassium ion and 1450 ppm fluoride, and to a control toothpaste with 1450 ppm fluoride: a three-day clinical study in New Jersey, USA. J Clin Dent. 2009;20(4):123-30.

71 - Ni LX, He T, Chang A, Sun L. The desensitizing efficacy of a novel stannous-containing sodium fluoride dentifrice: an 8-week randomized and controlled clinical trial. Am J Dent. 2010;23(Spec No B):17B-21B.

72 - Ong G, Strahan JD. Effect of a desensitizing dentifrice on dentinal hypersensitivity. Endod Dent Traumatol. 1989;5(5):213-8. doi: 10.1111/j.1600-9657.1989.tb00364.x

73 - Ongphichetmetha N, Lertpimonchai A, Champaiboon C. Bioactive glass and arginine dentifrices immediately relieved dentine hypersensitivity following non-surgical periodontal therapy: a randomized controlled trial. J Periodontol. 2021. doi: 10.1002/JPER.21-0091. Epub ahead of print

74 - Orsini G, Procaccini M, Manzoli L, Giuliodori F, Lorenzini A, Putignano A. A double-blind randomized-controlled trial comparing the desensitizing efficacy of a new dentifrice containing carbonate/hydroxyapatite nanocrystals and a sodium fluoride/potassium nitrate dentifrice. J Clin Periodontol. 2010;37(6):510-7. doi: 10.1111/j.1600-051X.2010.01558.x

75 - Orsini G, Procaccini M, Manzoli L, Sparabombe S, Tiriduzzi P, Bambini F, et al. A 3-day randomized clinical trial to investigate the desensitizing properties of three dentifrices. J Periodontol. 2013;84(11):e65-73. doi: 10.1902/jop.2013.120697

76 - Parkinson C, Hughes N, Jeffery P, Jain R, Kennedy L, Qaqish J, et al. The efficacy of an experimental dentifrice containing 0.454% w/w stannous fluoride in providing relief from the pain of dentin hypersensitivity: an 8-week clinical study. Am J Dent. 2013;26(Spec No A):25a-31a.

77 - Parkinson CR, Jeffery P, Milleman JL, Milleman KR, Mason S. Confirmation of efficacy in providing relief from the pain of dentin hypersensitivity of an anhydrous dentifrice containing 0.454% with or without stannous fluoride in an 8-week randomized clinical trial. Am J Dent. 2015;28(4):190-6.

78 - Parkinson CR, Hughes N, Hall C, Whelton H, Gallob J, Mason S. Three randomized clinical trials to assess the short-term efficacy of anhydrous 0.454% w/w stannous fluoride dentifrices for the relief of dentin hypersensitivity. Am J Dent. 2016;29(1):25-32.

79 - Parkinson C, Constantin P, Goyal C, Hall C. An exploratory clinical trial to evaluate the efficacy of an experimental dentifrice formulation in the relief of dentine hypersensitivity. J Dent. 2017;56:39-44. doi: 10.1016/j.jdent.2016.10.013

80 - Patel VR, Shettar L, Thakur S, Gillam D, Kamala DN. A randomised clinical trial on the efficacy of 5% fluorocalcium phosphosilicate-containing novel bioactive glass toothpaste. J Oral Rehabil. 2019;46(12):1121-6. doi: 10.1111/joor.12847

81 - Plagmann HC, Konig J, Bernimoulin JP, Rudhart AC, Deschner J. A clinical study comparing two high-fluoride dentifrices for the treatment of dentinal hypersensitivity. Quintessence Int. 1997;28(6):403-8.

82 - Pradeep A, Sharma A. Comparison of clinical efficacy of a dentifrice containing calcium sodium phosphosilicate to a dentifrice containing potassium nitrate and to a placebo on dentinal hypersensitivity: a randomized clinical trial. J Periodontol. 2010;81(8):1167-73. doi: 10.1902/jop.2010.100056

83 - Pradeep A, Agarwal E, Naik S, Bajaj P, Kalra N. Comparison of efficacy of three commercially available dentifrices [corrected] on dentinal hypersensitivity: a randomized clinical trial. Aust Dent J. 2012;57(4):429-34. doi: 10.1111/j.1834-7819.2012.01726.x

84 - Prasad KV, Sohoni R, Tikare S, Yalamalli M, Rajesh G, Javali SB. Efficacy of two commercially available dentifrices in reducing dentinal hypersensitivity. Indian J Dent Res. 2010;21(2):224-30. doi: 10.4103/0970-9290.66639

85 - Que K, Fu Y, Lin L, Hu D, Zhang YP, Panagakos FS, et al. Dentin hypersensitivity reduction of a new toothpaste containing 8.0% arginine and 1450 ppm fluoride: an 8-week clinical study on Chinese adults. Am J Dent. 2010;23(Spec No A):28a-25a.

86 - Salian S, Thakur S, Kulkarni S, LaTorre G. A randomized controlled clinical study evaluating the efficacy of two desensitizing dentifrices. J Clin Dent. 2010;21(3):82-7.

87 - Salvato AR, Clark GE, Gingold J, Curro FA. Clinical effectiveness of a dentifrice containing potassium chloride as a desensitizing agent. Am J Dent. 1992;5(6):303-6.

88 - Satyapal T, Mali R, Mali A, Patil V. Comparative evaluation of a dentifrice containing calcium sodium phosphosilicate to a dentifrice containing potassium nitrate for dentinal hypersensitivity: a clinical study. J Indian Soc Periodontol. 2014;18(5):581-5. doi: 10.4103/0972-124X.142447

89 - Schiff T, Bonta Y, Proskin HM, DeVizio W, Petrone M, Volpe AR. Desensitizing efficacy of a new dentifrice containing 5.0% potassium nitrate and 0.454% stannous fluoride. Am J Dent. 2000;13(3):111-5.

90 - Schiff T, Dotson M, Cohen S, De Vizio W, McCool J, Volpe A. Efficacy of a dentifrice containing potassium nitrate, soluble pyrophosphate, PVM/MA copolymer, and sodium fluoride on dentinal hypersensitivity: a twelve-week clinical study. J Clin Dent. 1994;5(Spec No):87-92.

91 - Schiff T, Santos M, Laffi S, Yoshioka M, Baines E, Brasil KD, et al. Efficacy of a dentifrice containing 5% potassium nitrate and 1500 PPM sodium monofluorophosphate in a precipitated calcium carbonate base on dentinal hypersensitivity. J Clin Dent. 1998;9(1):22-5.

92 - Schiff T, Zhang YP, DeVizio W, Stewart B, Chaknis P, Petrone ME, et al. A randomized clinical trial of the desensitizing efficacy of three dentifrices. Compend Cont Educ Dent. 2000;21(27):4-10. quiz 28.

93 - Schiff T, Saletta L, Baker RA, Winston JL, He T. Desensitizing effect of a stabilized stannous fluoride/sodium hexametaphosphate dentifrice. Compend Cont Educ Dent. 2005;26(9 Suppl 1):35-40.

94 - Schiff T, He T, Sagel L, Baker R. Efficacy and safety of a novel stabilized stannous fluoride and sodium hexametaphosphate dentifrice for dentinal hypersensitivity. J Contemp Dent Pract. 2006;7(2):1-8.

95 - Schiff T, Mateo L, Delgado E, Cummins D, Zhang Y, DeVizio W. Clinical efficacy in reducing dentin hypersensitivity of a dentifrice containing 8.0% arginine, calcium carbonate, and 1450 ppm fluoride compared to a dentifrice containing 8% strontium acetate and 1040 ppm fluoride under consumer usage conditions before and after switch-over. J Clin Dent. 2011;22(4):128-38.

96 - Seong J, Parkinson CP, Davies M, Claydon NCA, West NX. Randomised clinical trial to evaluate changes in dentine tubule occlusion following 4 weeks use of an occluding toothpaste. Clin Oral Invest. 2018;22(1):225-33. doi: 10.1007/s00784-017-2103-5

97 - Seong J, Newcombe RG, Matheson JR, Weddell L, Edwards M, West NX. A randomised controlled trial investigating efficacy of a novel toothpaste containing calcium silicate and sodium phosphate in dentine hypersensitivity pain reduction compared to a fluoride control toothpaste. J Dent. 2020;98:103320. doi: 10.1016/j.jdent.2020.103320

98 - Seong J, Newcombe RG, Foskett HL, Davies M, West NX. A randomised controlled trial to compare the efficacy of an aluminium lactate/potassium nitrate/hydroxylapatite toothpaste with a control toothpaste for the prevention of dentine hypersensitivity. J Dent. 2021;108:103619. doi: 10.1016/j.jdent.2021.103619

99 - Sharma N, Roy S, Kakar A, Greenspan D, Scott R. A clinical study comparing oral formulations containing 7.5% calcium sodium phosphosilicate (NovaMin), 5% potassium nitrate, and 0.4% stannous fluoride for the management of dentin hypersensitivity. J Clin Dent. 2010;21(3):88-92.

100 - Sharma D, McGuire JA, Gallob JT, Amini P. Randomised clinical efficacy trial of potassium oxalate mouthrinse in relieving dentinal sensitivity. J Dent. 2013;41(Suppl 4):S40-8. doi: 10.1016/S0300-5712(13)70005-8

101 - Sharma D, McGuire JA, Amini P. Randomized trial of the clinical efficacy of a potassium oxalate-containing mouthrinse in rapid relief of dentin sensitivity. J Clin Dent. 2013;24(2):62-7.

102 - Silverman G. The sensitivity-reducing effect of brushing with a potassium nitrate-sodium monofluorophosphate dentifrice. Compend Cont Educ Dent 1985;6(2):131-6.

103 - Silverman G, Gingold J, Curro FA. Desensitizing effect of a potassium chloride dentifrice. Am J Dent. 1994;7(1):9-12.

104 - Silverman G, Berman E, Hanna C, Salvato A, Fratarcangelo P, Bartizek RD. Assessing the efficacy of three dentifrices in the treatment of dentinal hypersensitivity. J Am Dent Assoc. 1996;127(2):191-201. doi: 10.14219/jada.archive.1996.0169

105 - Sowinski JA, Battista GW, Petrone ME, Chaknis P, Zhang YP, DeVizio W, et al. A new desensitizing dentifrice--an 8-week clinical investigation. Compend Cont Educ Dent. 2000;(27):11-16. quiz 28.

106 - Sowinski JA, Bonta Y, Battista GW, Chaknis P, Zhang YP, DeVizio W, et al. Desensitizing efficacy of Colgate Sensitive Maximum Strength and Fresh Mint Sensodyne dentifrices. Am J Dent. 2000;13(3):116-20.

107 - Sowinski J, Ayad F, Petrone M, DeVizio W, Volpe A, Ellwood R, et al. Comparative investigations of the desensitising efficacy of a new dentifrice. J Clin Periodontol. 2001;28(11):1032-36. doiI: 10.1034/j.1600-051x.2001.281107.x

108 - Sowinski JA, Kakar A, Kakar K. Clinical evaluation of the Jay Sensitivity Sensor Probe: a new microprocessor-controlled instrument to evaluate dentin hypersensitivity. Am J Dent. 2013;26(Spec No B):5B-12B.

109 - Sufi F, Hall C, Mason S, Shaw D, Kennedy L, Gallob JT. Efficacy of an experimental toothpaste containing 5% calcium sodium phosphosilicate in the relief of dentin hypersensitivity: an 8-week randomized study (Study 1). Am J D Dent. 2016;29(2):93-100.

110 - Sufi F, Hall C, Mason S, Shaw D, Milleman J, Milleman K. Efficacy of an experimental toothpaste containing 5% calcium sodium phosphosilicate in the relief of dentin hypersensitivity: an 8-week randomized study (Study 2). Am J Dent. 2016;29(2):101-9.

111 - Surve SM, Acharya AB, Shetty A, Thakur SL. Efficacy of calcium sodium phosphosilicate in managing dentinal hypersensitivity. Gen Dent. 2012;60(5):e308-11.

112 - Tao D, Ling MR, Feng XP, Gallob J, Souverain A, Yang W, et al. Efficacy of an anhydrous stannous fluoride toothpaste for relief of dentine hypersensitivity: a randomized clinical study. J Clin Periodontol. 2020;47(8):962-9. doi: 10.1111/jcpe.13305

113 - Tarbet WJ, Silverman G, Stolman JM, Fratarcangelo PA. An evaluation of two methods for the quantitation of dentinal hypersensitivity. J Am Dent Assoc. 1979;98(6):914-8.

114 - Tarbet W, Silverman G, Stolman J, Fratarcangelo P. Clinical evaluation of a new treatment for dentinal hypersensitivity. J Periodontol. 1980;51(9):535-40. doi: 10.1902/jop.1980.51.9.535

115 - Tarbet W, Silverman G, Fratarcangelo P, Kanapka J. Home treatment for dentinal hypersensitivity: a comparative study. J Am Dent Assoc. 1982;105(2):227-30. doi: 10.14219/jada.archive.1982.0092

116 - Vano M, Derchi G, Barone A, Covani U. Effectiveness of nano-hydroxyapatite toothpaste in reducing dentin hypersensitivity: a double-blind randomized controlled trial. Quintessence Int. 2014;45(8):703-11. doi: 10.3290/j.qi.a32240

117 - Vano M, Derchi G, Barone A, Pinna R, Usai P, Covani U. Reducing dentine hypersensitivity with nano-hydroxyapatite toothpaste: a double-blind randomized controlled trial. Clin Oral Invest. 2018;22(1):313-20. doi: 10.1007/s00784-017-2113-3

118 - Wara-aswapati N, Krongnawakul D, Jiraviboon D, Adulyanon S, Karimbux N, Pitiphat W. The effect of a new toothpaste containing potassium nitrate and triclosan on gingival health, plaque formation and dentine hypersensitivity. J Clin Periodontol. 2005;32(1):53-8. doi: 10.1111/j.1600-051X.2004.00631.x

119 - West NX, Addy M, Jackson RJ, Ridge DB. Dentine hypersensitivity and the placebo response. A comparison of the effect of strontium acetate, potassium nitrate and fluoride toothpastes. J Clin Periodontol. 1997;24(4):209-15. doi: 10.1111/j.1600-051x.1997.tb01833.x

120 - West N, Newcombe R, Hughes N, Mason S, Maggio B, Sufi F, et al. A 3-day randomised clinical study investigating the efficacy of two toothpastes, designed to occlude dentine tubules, for the treatment of dentine hypersensitivity. J Dent. 2013;41(2):187-94. doi: 10.1016/j.jdent.2012.11.007

121 - Xia Y, Yang ZY, Li YH, Zhou Z. The Effects of a toothpaste containing the active ingredients of galla chinensis and sodium fluoride on dentin hypersensitivity and sealing of dentinal tubules: an in vitro study and an eight-week clinical study in 98 patients. Med Sci Monit. 2020;26:e920776. doi: 10.12659/MSM.920776

122 - Yates R, Ferro R, Newcombe RG, Addy M. A comparison of a reformulated potassium citrate desensitising toothpaste with the original proprietary product. J Dent. 2005;33(1):19-25. doi: 10.1016/j.jdent.2004.07.004

123 - Young S, Sufi F, Siddiqi M, Maclure R, Holt J. A randomized non-inferiority clinical study to evaluate the efficacy of two dentifrices for dentin hypersensitivity. J Clin Dent. 2016;27(4):97-104.

124 - Young S, Wang N, Mason S, Sufi F. A randomized clinical study to evaluate the efficacy of an experimental 3.75% (w/w) potassium chloride dentifrice for the relief of dentin hypersensitivity. J Clin Dent. 2017;28(2):9-15.

125 - Zhang JZ, Wang LN, Liu QC. Effect of potassium toothpaste on treatment of tooth essence allergy. J Dalian Med Univ. 2010;32(3):312-3.

126 - Zang P, Parkinson C, Hall C, Wang N, Jiang H, Zhang J, et al. A randomized clinical trial investigating the effect of particle size of calcium sodium phosphosilicate (CSPS) on the efficacy of csps-containing dentifrices for the relief of dentin hypersensitivity. J Clin Dent. 2016;27(2):54-60.

127 - Zang P, Shaw D. A randomized clinical study to evaluate the efficacy of an 8% (w/w) strontium acetate dentifrice in providing relief from dentinal hypersensitivity. J Clin Dent. 2016;27(4):91-6.

128 - Zhuang W, Cao D. Efficacy of a dentifrice containing 5% potassium nitrate on dental hypersensitivity. Shanghai J Stomatol. 2011;20(6):638-40.

129 - GSK Clinical Trials, GlaxoSmithKline, investigators and sponsors. Short term clinical study investigating the efficacy of an occluding dentifrice in providing relief from dentinal hypersensitivity. ClinicalTrials.gov [Internet]. Bethesda (United States): U.S. National Library of Medicine; 2016 [cited 2021 Dec 15]. Available from: https://clinicaltrials.gov/ct2/show/NCT02705716?term=NCT02705716

130 - Lunch M, investigator; Johnson & Johnson Consumer and Personal Products Worldwide, sponsor. A test on a new experimental mouth rinse for relieving tooth sensitivity. ClinicalTrials.gov [Internet]. Bethesda (United States): U.S. National Library of Medicine; 2012 [cited 2021 Dec 15]. Available from: https://clinicaltrials.gov/ct2/show/NCT01727258

131 - GSK Clinical Trials, GlaxoSmithKline, investigators and sponsors. A proof of concept study to evaluate the efficacy of an occlusion based dentifrice in the relief of dentinal hypersensitivity. ClinicalTrials.gov [Internet]. Bethesda (United States): U.S. National Library of Medicine; 2012 [cited 2021 Dec 15]. Available from: https://clinicaltrials.gov/ct2/show/NCT01691560

132 - GSK Clinical Trials, GlaxoSmithKline, investigators and sponsors. An exploratory clinical study to evaluate the efficacy of two currently marketed toothpastes containing different concentrations of a tubule occlusion agent. ClinicalTrials.gov [Internet]. Bethesda (United States): U.S. National Library of Medicine; 2012 [cited 2021 Dec 15]. Available from: https://www.clinicaltrials.gov/ct2/show/NCT01075256

133 - Syed Jaffar Abbas Zaidi, investigator; Dow University of Health Sciences, sponsor. Immediate and sustained treatment response of commercially available biomin f, colgate sensitive pro-relief and sensodyne rapid action dentifrices in dentin hypersensitivity - a randomized clinical trial. ClinicalTrials.gov [Internet]. Bethesda (United States): U.S. National Library of Medicine; 2019 [cited 2021 Dec 15]. Available from: https://clinicaltrials.gov/ct2/show/NCT04249336

134 - GSK Clinical Trials, GlaxoSmithKline, investigators and sponsors. Randomized controlled examiner-blind phase ii exploratory clinical study to characterize the efficacy profile of an experimental dual active combination dentifrice for the relief of dentin hypersensitivity, in subjects with clinically diagnosed dentin hypersensitivity. ClinicalTrials.gov [Internet]. Bethesda (United States): U.S. National Library of Medicine; 2019 [cited 2021 Dec 15]. Available from: https://clinicaltrials.gov/ct2/show/NCT03943095

135 - GSK Clinical Trials, GlaxoSmithKline, investigators and sponsors. A clinical study investigating the efficacy of a dentifrice in providing long term relief from dentinal hypersensitivity. ClinicalTrials.gov [Internet]. Bethesda (United States): U.S. National Library of Medicine; 2012 [cited 2021 Dec 15]. Available from: https://clinicaltrials.gov/ct2/show/NCT01592864

136 - Procter and Gamble, investigators and sponsors. A randomized clinical study to assess the effects of various dentifrice technologies on dentinal hypersensitivity. ClinicalTrials.gov [Internet]. Bethesda (United States): U.S. National Library of Medicine; 2019 [cited 2021 Dec 15]. Available from: https://clinicaltrials.gov/ct2/show/NCT03965039

137 - GSK Clinical Trials, GlaxoSmithKline, investigators and sponsors. Short term clinical study investigating the efficacy of an occluding dentifrice in providing relief from dentinal hypersensitivity. ClinicalTrials.gov [Internet]. Bethesda (United States): U.S. National Library of Medicine; 2016 [cited 2021 Dec 15]. Available from: https://clinicaltrials.gov/ct2/show/NCT02773758

138 - Amaechi BT, investigator; University of Texas Health Science Center San Antonio Clinical, sponsor. Clinical efficacy in relieving dentin hypersensitivity of nanohydroxyapatite-containing toothpastes and cream. ClinicalTrials.gov [Internet]. Bethesda (United States): U.S. National Library of Medicine; 2015 [cited 2021 Dec 15]. Available from: https://clinicaltrials.gov/ct2/show/NCT02918617
