# Supplementary material for: Formulations of desensitizing toothpastes for dentin hypersensitivity: a scoping review
Source: J Appl Oral Sci. 2022 Feb 7;30:e20210410. doi: 10.1590/1678-7757-2021-0410 (PMC8908863; doi:10.1590/1678-7757-2021-0410)
Supplement: Supplementary file 2 [file 1678-7757-jaos-30-e20210410-app02.pdf]

Systematic Review
http://dx.doi.org/10.1590/1678-7757-2021-0410

JAOS
JOURNAL OF APPLIED ORAL SCIENCE

Appendix 2 - Studies excluded (with reasons) after full-text analysis

	Study	Reason for exclusion
1.	Acceptance of Sensodyne toothpaste for sensitive teeth. Council on Dental Therapeutics. J Am Dent Assoc. 1985;110(3):394-5. doi: 10.14219/jada.archive.1985.0318.	A letter to the editor with original data which fails to report numerical outcome data. It is impossible to extract data.
2.	Acceptance of promise with fluoride and Sensodyne-F toothpastes for sensitive teeth. Council on Dental Therapeutics. J Am Dent Assoc. 1986;113(4):673-5. doi: 10.14219/jada.archive.1986.0244	Letter to the editor. Formulas and methods are unclear. The study lacks enough information to be abstracted.
3.	Toothpaste becomes first OHF approved product for relief of tooth sensitivity and remineralisation. Br Dent J. 2019;226:233. doi: 10.1038/sj.bdj.2019.123	Editorial.
4.	Addy M, Mostafa P, Newcombe RG. Effect of plaque of five toothpastes used in the treatment of dentin hypersensitivity. Clin Prev Dent. 1990;12:28-33.	The article fails to clinically examine DH (thermal, cold or tactil stimulus).
5.	Aguas SC, Policicchio JE. Valoración clínica de una pasta dentífica desensibilizante [Clinical evaluation of a desensitizing toothpaste]. Rev del Círculo Argentino Odontol. 1970;33:43-6. Spanish	Unaccessed.
6.	Alaskar NA. Comparative effectiveness research of dentin hypersensitivity intervention - a mixed systematic review analysis. Los Angeles: University of California; 2016. 89 p.	Systematic review.
7.	Antoniazzi RP, Machado ME, Grellmann AP, Santos RC, Zanatta FB. Effectiveness of a desensitizing agent for topical and home use for dentin hypersensitivity: a randomized clinical trial. Am J Dent. 2014;27:251-7.	The study lacks a comparison group.
8.	Aparna S, Setty S, Thakur S. Comparative efficacy of two treatment modalities for dentinal hypersensitivity: a clinical trial. Indian J Dent Res. 2010;21(4):544-8. doi: 10.4103/0970-9290.74213	The study lacks a comparison group.
9.	Ayad F, Vandeven M, Cummins D, Delgado E, Zhang Y, Devizio W. Dentin hypersensitivity reduction efficacy of a dentifrice containing 5% KNO3. In: Proceedings of the 41st Annual Meeting & Exhibition of the American Association for Dental Research & the 36th Annual Meeting of the Canadian Association for Dental Research; 2012. Abstract: 1539.	Conference abstract.
10.	Ayad F, Ayad N, Vazquez J, Zhang YP, Mateo LR, Cummins D. Use of a toothpaste containing 8% arginine and calcium carbonate for immediate and lasting relief of dentin hypersensitivity: a simple and effective in-office procedure. Am J Dent. 2018;31(3):135-40.	This single-arm study lacks a comparison group.
11.	Bacić M, Sutalo J, Lincir I. Klinicko ispitivanje stroncij klorida u lijecenju dentinske preosjetljivosti [Strontium chloride in the treatment of dental hypersensitivity]. Acta Stomatol Croat. 1987;21(2):93-100. Croatian	The study lacks details on outcome measurement, i.e, patients reporting improvement, and apparently uses a nonvalidated scale.
12.	Bae JH, Kim YK, Myung SK. Desensitizing toothpaste versus placebo for dentin hypersensitivity: a systematic review and meta-analysis. J Clin Periodontol. 2015;42(2):131-41. doi: 10.1111/jcpe.12347	Systematic review.
13.	Blitzer B. Evaluación del cloruro de estroncio en el tratamiento de la sensibilidad dentaria post-tratamiento periodontal [Evaluation of the use of strontium chloride in the treatment of tooth sensitivity following periodontal therapy]. Rev Asoc Odontol Argent. 1966;54(1):13-4. Spanish	The study fails to report DH clinical diagnosis.
14.	Blitzer B. A consideration of the possible causes of dental hypersensitivity: treatment by a strontium-ion dentifrice. Periodontics. 1967;5(6):318-21.	The study fails to report DH clinical diagnosis.
15.	Blitzer B. Una consideraciõn sobre las posibles causas de la hipersensibilidad dental: tratamiento por un dentifrico con ion-estroncio [Possible causes of tooth hypersensitivity: treatment with a strontium-ion dentifrice]. Rev Odontol Ecuat. 1972;17(54):1-4. Spanish	The study fails to report DH clinical diagnosis.

16.	Blitzwe B. Una consideración sobre las posibles causas de la hipersensibilidad dental: tratamiento por un dentifrico con ion-estroncio [Possible causes of dental hypersensitivity: treatment with a strontium-ion dentifrice]. Rev Dent (San Salv). 1970;17(49):31-6. Spanish	The study fails to report DH clinical diagnosis.
17.	B Low S, Allen EP, Kontogiorgos ED. Reduction in dental hypersensitivity with nano-hydroxyapatite, potassium nitrate, sodium monoflurophosphate and antioxidants. Open Dent J. 2015;(9):92-7. doi: 10.2174/1874364101509010092	The study lacks a comparison group and reports on clinical diagnosis by thermal, cold or tactil stimuli.
18.	Bm S, P P, Sanghani NN. Chair side application of NovaMin for the treatment of dentinal hypersensitivity- a novel technique. J Clin Diagn Res. 2014;8(10):ZC05-8. doi: 10.7860/JCDR/2014/8824.4947	In-office treatment after periodontal treatment.
19.	Bolden TE. A desensitizing dentifrice with multiple oral health benefits formulated for daily use. J Clin Dent.1994;5 Spec No:68-70.	Literature review.
20.	Brook A, Francis C, Joshi R, Reekie R, Bransbury D. Efficacy of a potassium nitrate toothpaste in dentinal hypersensitivity. J Dent Res. 1994;73.	Conference abstract.
21.	Muzzin KB, Johnson R. Effects of potassium oxalate on dentin hypersensitivity in vivo. J Periodontol. 1989;60(3):151-8. doi: 10.1902/jop.1989.60.3.151	Conference abstract; teeth received different treatments; split-mouth design.
22.	Browning WD, Cho SD, Deschepper EJ. Effect of a nano-hydroxyapatite paste on bleaching-related tooth sensitivity. J Esthet Restor Dent. 2012;24(4):268-76. doi: 10.1111/j.1708-8240.2011.00437.x	Use of toothpaste for bleaching.
23.	Bubani G. Desnesibilizzazione dentinale con un nuovo gel fluorurato [Dentin desensitization with a new fluoride-containing gel]. Riv Odontostomatol Implantoprotesi. 1983;(2):81-2. Italian	Unaccessed.
24.	Carrasco HP. Strontium chloride toothpaste--effectiveness as related to duration of use. Pharmacol Ther Dent. 1971;1(4):209-15.	The study lacks a description of the clinical diagnosis for tactile, air or cold stimulus. Thus, diagnosis is unclear.
25.	Carrasco HP. Dentrifrício com cloreto de estrôncio. Eficácia relacionada com o tempo de emprego [Dentifrice with strontium chloride. Efficiency related to time of application]. Quintessencia. 1979;6(2):48-54. Portuguese	The study lacks a description of the clinical diagnosis for tactile, air or cold stimulus. Thus, diagnosis is unclear.
26.	Carson SJ. Possible role for arginine-containing toothpastes in managing dentine hypersensitivity. Evid Based Dent. 2013;14(2):44-5. doi: 10.1038/sj.ebd.6400930	Letter to the editor.
27.	Champaiboon C, Lertpimonchai A, Lertpimonchai K. Bioactive glass versus Arginine dentifrices on the reduction of dentin permeability and acid tolerance. Clin Exp Dent Res. 2021;7(4):620-7. doi: 10.1002/cre2.372	*In-vitro* study.
28.	Chałas R, Wójcik-Chęcińska I, Zamościńska J, Bachanek T. Assessment of pain intensity in patients with dentin hypersensitivity after application of prophylaxis paste based on calcium sodium phosphosilicate formula. Med Sci Monit. 2015;21:2950-5. doi: 10.12659/MSM.894189	In-office treatment.
29.	Charig AJ, Thong S, Flores F, Gupta S, Major E, Winston AE. Mechanism of action of a desensitizing fluoride toothpaste delivering calcium and phosphate ingredients in the treatment of dental hypersensitivity. Part II: comparison with a professional treatment for tooth hypersensitivity. Compend Contin Educ Dent. 2009;30(9):622-4, 626, 628.	Animal study.
30.	Chesters R, Kaufman HW, Wolff MS, Huntington E, Kleinberg I. Use of multiple sensitivity measurements and logit statistical analysis to assess the effectiveness of a potassium-citrate-containing dentifrice in reducing dentinal hypersensitivity. J Clin Periodontol. 1992;19(4):256-61. doi:10.1111/j.1600-051x.1992.tb00463.x	The study lacks randomization.
31.	Chu CH, Lo EC. Immediate post-application effect of professional prophylaxis with 8% arginine-calcium carbonate desensitizing paste on hypersensitive teeth. a practitioner-based clinical trial. Am J Dent. 2014;27(1):7-11	Periodontal patients received in-office treatment.
32.	Ciancio SG. Chemical agents: plaque control, calculus reduction and treatment of dentinal hypersensitivity. Periodontol 2000. 1995;8:75-86. doi: 10.1111/j.1600-0757.1995.tb00046.x	Literature review.
33.	Clark DC, Hanley JA, Geoghegan S, Vinet D. The effectiveness of a fluoride varnish and a desensitizing toothpaste in treating dentinal hypersensitivity. J Periodontal Res. 1985;20(2):212-9. doi: 10.1111/j.1600-0765.1985.tb00428.x	The study lacks a report on clinical examinations by thermal, cold or air stimulus.

34.	Collins JF, Gingold J, Stanley H, Simring M. Reducing dentinal hypersensitivity with strontium chloride and potassium nitrate. Gen Dent. 1984;32(1):40-3.	Patients underwent periodontal surgery before treatment.
35.	Collins JR, Richardson D, Sotero K, Mateo LR, Mauriz I. Beneficial effects of an arginine-calcium carbonate desensitizing paste for treatment of dentin hypersensitivity. Am J Dent. 2013;26(2):63-7.	In-office treatment.
36.	Cooley RL, Sandoval VA. Effectiveness of potassium oxalate treatment on dentin hypersensitivity. Gen Dent. 1989;37(4):330-3.	In-office treatment lacking toothpastes.
37.	Cooper TM. Treatment of cervical hypersensitivity. J Tenn State Dent Assoc. 1968;48(4):277-81.	Letter to the editor.
38.	Cummins D. Clinical evidence for the superior efficacy of a dentifrice containing 8.0% arginine and calcium carbonate in providing instant and lasting relief of dentin hypersensitivity. J Clin Dent. 2011;22(4):97-9.	Letter to the editor.
39.	Cunha-Cruz J, Stout JR, Heaton LJ, Wataha JC, Northwest P. Dentin hypersensitivity and oxalates: a systematic review. J Dent Res. 2011;90(3):304-10. doi: 10.1177/0022034510389179.	Systematic review.
40.	Dabas VK, Swadia UH. Comparative study of effectiveness of strontium chloride and formalin containing dentifrices in relieving dental hypersensitivity. Indian J Dent Res. 1989;1(1):15-21.	The study tests formalin (not one of the investigated compounds) and lacks randomization.
41.	de Melo Alencar C, de Paula BL, Guanipa Ortiz MI, Barauna Magno M, Martins Silva C, Cople Maia L. Clinical efficacy of nano-hydroxyapatite in dentin hypersensitivity: a systematic review and meta-analysis. J Dent. 2019;82:11-21. doi: 10.1016/j.jdent.2018.12.014	Systematic review of nano-hydroxyapatite treatment.
42.	de Rabbione MR, Monteverde J. Dentifricos con cloruro de estroncio [Dentifrices with strontium chloride]. Odontoiatria]. Rev Iberoam Med Boca. 1965;19(219):137-40. Spanish	The study lacks a comparison group.
43.	Devji T. Dentifrice containing potassium chloride may be more effective than standard fluoride dentifrice for the relief of dentin hypersensitivity. J Am Dent Assoc. 2018;149(1):e11.doi: 10.1016/j.adaj.2017.09.037	Editorial
44.	Ding PH, Dai A, Hu HJ, Huang JP, Liu JM, Chen LL. Efficacy of nano-carbonate apatite dentifrice in relief from dentine hypersensitivity following non-surgical periodontal therapy: a randomized controlled trial. BMC Oral Health. 2020;12;20(1):170. doi: 10.1186/s12903-020-01157-9	Patients showing periodontal disease were subjected to non-surgical periodontal treatment, receiving, at their first consultation, in-office rubber cup polishing with the assigned toothpaste.
45.	Douglas de Oliveira DW, Oliveira ES, Mota AF, Pereira VH, Bastos VO, Glória JC, et al. Effectiveness of three desensitizing dentifrices on cervical dentin hypersensitivity: a pilot clinical trial. J Int Acad Periodontol. 2016;18(2):57-65.	In-office treatment with a split mouth design.
46.	Drake MA, Lenton PA, Lunos AS. A randomized clinical trial on the efficacy of a new oxalate-containing sensitivity relief strip following professional vs self-application. Int J Dent Hyg. 2018;16(2):e79-e87. doi: 10.1111/idh.12317	The ingredient was applied with strips.
47.	Elias Boneta AR, Ramirez K, Naboa J, Mateo LR, Stewart B, Panagokos F, et al. Efficacy in reducing dentine hypersensitivity of a regimen using a toothpaste containing 8% arginine and calcium carbonate, a mouthwash containing 0.8% arginine, pyrophosphate and PVM/MA copolymer and a toothbrush compared to potassium and negative control regimens: an eight-week randomized clinical trial. J Dent. 2013;41:S42-9. doi: 10.1016/j.jdent.2012.11.011	The comparison group evaluated other treatment than toothpastes.
48.	França IL, Sallum EA, Do Vale HF, Casati MZ, Sallum AW, Stewart B. Efficacy of a combined in-office/home-use desensitizing system containing 8% arginine and calcium carbonate in reducing dentin hypersensitivity: an 8-week randomized clinical study. Am J Dent. 2015;28(1):45-50.	In-office and home treatment in periodontal.
49.	Freda NM, Veitz-Keenan A. Calcium sodium phosphosilicate had some benefit on dentine hypersensitivity. Evid Based Dent. 2016;17(1):12-3. doi: 10.1038/sj.ebd.6401148	Letter to the editor.
50.	Gafar M, Stein M. Folosirea pastelor de dinţi şi a apelor de gură ca mijloc complementar în tratamentul hiperesteziei dentinare cu localizare în regiunea cervicală a dinţilor [Use of toothpastes and mouthwashes as supplementary aids in the treatment of dentin hyperesthesia localized in the cervical region of the teeth]. Rev Chir Oncol Radiol O R L Oftalmol Stomatol Ser Stomatol. 1975;22(1):35-40. Romanian.	The study lacked randomization.
51.	Gagliani N. Il trattamento ASBA nella terapia della ipersensibilità dentinale [ASBA therapy in the treatment of dentin hypersensitivity]. Rass Int di Stomatol Prat. 1967;18(5):345-54. Italian.	Case series.

52.	Garcia-Godoy F. A dentifrice with multiple benefits. Am J Dent. 2011;24:Spec No A:2A.	Letter to the editor.
53.	Garcia-Godoy F. An anhydrous stannous fluoride dentifrice for treating dentin hypersensitivity. Am J Dent. 2013;26:Spec No A:2A.	Letter to the editor.
54.	Gedalia I, Breyer L, Stabholz A, Shapira L. Clinical evaluation of the effectiveness of aminfluoride fluid, aminfluoride gelee, and strontiumchloride paste in relieving dentine sensitivity. Pharm Acta Helv. 1987;62(7):188-90.	Excluded: there is no comparison group with other toothpaste.
55.	Gillam DG, Weiss G, Bulman JS, Newman H. Efficacy of a novel Bioglass® dentifrice: results of an 8-week study. Int Dent J. 2000;50:355.	Excluded: conference abstract.
56.	Gillam DG. Clinical trial designs for testing of products for dentine hypersensitivity-a review. J West Soc Periodontol Abstr. 1997;45(2):37-46.	Excluded: literature review.
57.	Green BI, Green MI, McFall WT Jr. Calcium hydroxide and potassium nitrate as desensitizing agents for hypersensitive root surfaces. J Periodontol. 1977;48(10):667-72. doi: 10.1902/jop.1977.48.10.667	Excluded: there is no comparison with other toothpaste.
58.	Green ML, Green BL. Calcium hydroxide: an effective desensitizing agent. Dent Hyg (Chic). 1978;52(6):280-5.	Excluded: there is no comparison with other toothpaste.
59.	Hamlin D, Williams KP, Delgado E, Zhang YP, DeVizio W, Mateo LR. Clinical evaluation of the efficacy of a desensitizing paste containing 8% arginine and calcium carbonate for the in-office relief of dentin hypersensitivity associated with dental prophylaxis. Am J Dent. 2009;22 Spec No A:16A-20A.	Excluded: toothpaste compared to in-office dental prophilaxis.
60.	Hazen SP, Volpe AR, King WJ. Comparative desensitizing effect of dentifrices containing sodium monofluorophosphate, stannous fluoride, and formalin. Periodontics. 1968;6(5):230-2.	Excluded: DH was not evaluated by a clinical examination (thermal, cold or tactil stimulus).
61.	Hirsiger C, Schmidlin PR, Michaelis M, Hirsch C, Attin T, Heumann C, et al. Efficacy of 8% arginine on dentin hypersensitivity: A multicenter clinical trial in 273 patients over 24 weeks. J Dent. 2019;83:1-6. doi: 10.1016/j.jdent.2019.01.002	Excluded: in office study.
62.	Hodosh M. A superior desensitizer--potassium nitrate. J Am Dent Assoc. 1974;88(4):831-2. doi: 10.14219/jada.archive.1974.0174	Excluded: there is no comparison group to other toothpaste.
63.	Hodosh M. Current treatment for dentinal hypersensitivity. Potassium nitrate as a desensitizer. Compend Contin Educ Dent. 1982;S120-1.	Letter to the editor.
64.	Hodosh M. Potassium nitrate. Gen Dent. 2000;48(1):12.	Letter to the editor.
65.	Hodosh M. Potentiating potassium nitrate's desensitization with dimethyl isosorbide. Gen Dent. 2001;4(5):531-6.	Excluded: the methods is unclear related to time of follow up, statistical methods, type of treatment (if it is toothpaste or in-office treatment). The study was conducted in private practice and it does not seem be a RCT.
66.	Hüttemann R, Dönges H. Untersuchungen zur Therapie überempfindlicher Zahnhälse mit Hydroxylapatit [Treatment of dentine hypersensitivity with hydroxylapatite]. Dtsch Zahnarztl Z. 1987;42(5): 486-8. German.	Excluded: the data can not be extracted. The study lacks information: there is no baseline information, no mean values reported, no sample in each group provided.
67.	Jena A, Shashirekha G. Comparison of efficacy of three different desensitizing agents for in-office relief of dentin hypersensitivity: a 4 weeks clinical study. J Conserv Dent. 2015;18(5):389-93. doi: 10.4103/0972-0707.164052	Excluded: in-office treatment.
68.	Jiang H, Minquan DU. Efficacy of a dentifrice containing 5.53% potassium citrate on dentinal hypersensitivity. J Oral Sci Res. 2010;26:732-4	Excluded: not found. Manual search.
69.	Johnson RH, Zulgar-Nain BJ, Koval JJ. The effectiveness of an electro-ionizing toothbrush in the control of dentinal hypersensitivity. J Periodontol. 1982;53(6):353-9.doi: 10.1902/jop.1982.53.6.353	Excluded: electrical toothbrush.
70.	Kandelman D, Gagnon G, Ruel D, Peters D, Trepanier J. Evaluation clinique de l'action de deux dentifrices [Clinical evaluation of the effect of 2 high fluoride content dentifrices on dentin hypersensitivity]. Inf Dent. 1990;72(38):3609-14. French.	Excluded: not random; the diagnosis is unclear.
71.	Kanouse MC, Ash MM-Jr. The effectiveness of a sodium monofluorophosphate dentifrice on dental hypersensitivity. J Periodontol. 1969;40(1):38-40. doi: 10.1902/jop.1969.40.1.38	Excluded: it is not a randomized controlled trial.

72.	Kapferer I, Pflug C, Kisielewsky I, Giesinger J, Beier US, Dumfahrt H. Instant dentin hypersensitivity relief of a single topical application of an in-office desensitizing paste containing 8% arginine and calcium carbonate: a split-mouth, randomized-controlled study. Acta Odontol Scand. 2013;71(3-4):994-9. doi: 10.3109/00016357.2012.741701	Excluded: in-office treatment and split mouth design.
73.	Karim BF, Gillam DG. The efficacy of strontium and potassium toothpastes in treating dentine hypersensitivity: a systematic review. Int J Dent. 2013;2013:573258. doi: 10.1155/2013/573258	Excluded: systematic review.
74.	Kaufman HW, Wolff MS, Winston AE, Triol CW. Clinical evaluation of the effect of a remineralizing toothpaste on dentinal sensitivity. J Clin Dent. 1999;10:50-4.	Excluded: is not a randomized trial.
75.	Keil E, Tschamer H. Behandlung von hypersensiblem Dentin mit jodhaltiger Zahnpasta [Treatment of hypersensitive dentin with iodine-containing toothpaste]. Zahnarztl Welt Zahnarztl Rundsch ZWR Zahnarztl Reform. 1966;67(9):325-6. German	Excluded: it is not a randomized trial.
76.	Knupfer WH. [Cervical tooth hypersensitivity. Clinical-experimental results with a desensitizing toothpaste]. Riv Odontostomatol Implantoprotesi. 1984; 93(5):95-6. [In Italian]	Excluded: not accessed.
77.	Konekeri V, Bennadi D, Manjunath M, Kshetrimayum N, Siluvai S, Reddy CV. A clinical study to assess the effectiveness of CPP-ACP (casein phosphopeptide-amorphous calcium phosphate) versus potassium-nitrate (KNO3) on cervical dentine hypersensitivity. J Young Pharmacists. 2015;7:217-24.	Excluded: the comparsion group is gel, not toothpaste.
78.	Kumar S, Thomas BS, Gupta K, Guddattu V, Alexander M. Iontophoresis and topical application of 8% arginine-calcium carbonate to treat dentinal hypersensitivity. Niger J Clin Pract. 2018;21(8):1029-33. doi: 10.4103/njcp.njcp_341_17	Excluded: comparison group was iontophoresis.
79.	Lamont T, Innes N. Study suggests dentine bonding agents provided better relief from dentine hypersensitivity than a desensitising toothpaste. Evid Based Dent. 2013;14(4):105-6. doi: 10.1038/sj.ebd.6400965	Excluded: editorial.
80.	Layer TM. Development of a fluoridated, daily-use toothpaste containing NovaMin technology for the treatment of dentin hypersensitivity. J Clin Dent. 2011;22(3):59-61.	Excluded: editorial.
81.	Lecointre C, Apiou J, Marty P, Poitou P. Controlled trial of the action of a toothpaste containing nicomethanol hydrofluoride in the treatment of dentine hypersensitivity. J Int Med Res. 1986;14(4):217-22. doi: 10.1177/030006058601400409	Excluded: the comparison group is not a current group of interset.
82.	Leight RS, Sufi F, Gross R, Mason SC, Barlow AP. Dentinal hypersensitivity: a 12-week study of a novel dentifrice delivery system comparing different brushing times and assessing the efficacy for hard-to-reach molar teeth. J Clin Dent. 2008; 19(4):147-53.	Excluded: there is no comparison with other toothpaste group.
83.	Leous P, Rudenkova N, Bourgeois D. Clinical evaluation of the desentizing of toothpaste with a high content of fluoride in patients with dentine hypersensitivity. J Dent Res. 1999;78:356, Abstract 2003.	Excluded: conference abstract, there is no comparison with other toothpaste group.
84.	Levenson D. Beneficial effects seen with most desensitising toothpastes. Evid Based Dent. 2016;17(1):10-1. doi: 10.1038/sj.ebd.6401147	Excluded: editorial.
85.	Levin MP, Yearwood LL, Carpenter WN. The desensitizing effect of calcium hydroxide and magnesium hydroxide on hypersensitive dentin. Oral Surg Oral Med Oral Pathol. 1973;35(5):741-6. doi: 10.1016/0030-4220(73)90044-3	Excluded: in-office treatment.
86.	Li R, Tang XJ, Li YH, Wang Y, Chen J, Wang QH, et al. Arginine-containing desensitizing toothpaste for dentine hypersensitivity: a meta-analysis. Chin J Evid-Based Med. 2011;11:570-5.	Excluded: systematic review.
87.	Magno MB, Nascimento GC, Da Penha NK, Pessoa OF, Loretto SC, Maia LC. Difference in effectiveness between strontium acetate and arginine-based toothpastes to relieve dentin hypersensitivity. A systematic review. Am J Dent. 2015;28(1):40-4.	Excluded: systematic review.
88.	Mahesuti A, Duan YI, Wang G, Cheng XR, Matis BA. Short-term efficacy of agents containing KNO3 or CPP-ACP in treatment of dentin hypersensitivity. Chin J Dent Res. 2014;17(1):43-7.	Excluded: the comparison group is gel, not toothpaste.

89.	Makeeva IM, Polyakova MA, Avdeenko OE, Paramonov YO, Kondrat'ev AS, Pilyagina AA. Otsenka effektivnosti dlitel'nogo primeneniya zubnoi pasty Apadent Total Care, soderzhashchei Meditsinskii nano-gidroksiapatit [Effect of long term application of toothpaste Apadent Total Care Medical nano-hydroxyapatite]. Stomatologiia (Mosk). 2016;95(4):34-6. Russian. doi: 10.17116/stomat201695434-36	Excluded: no randomization is mentioned; the study uses only one toothpaste (nano calcium hydroxyapatite) as the case group, but does not report a different treatment for the control group.
90.	Makeeva IM, Polyakova MA, Doroshina VY, Sokhova IA, Arakelyan MG, Makeeva MK. Éffektivnost' vliianiia pasty i suspenzii s nano-gidroksiapatitom na chuvstvitel'nost' zubov pri retsessii desny [Efficiency of paste and suspension with nano-hydroxyapatite on the sensitivity of teeth with gingival recession]. Stomatologiia (Mosk). 2018;97(4):23-7. Russian. doi: 10.17116/stomat20189704123	Excluded: the study compared suspension. There is no toothpaste as a test group.
91.	Manocher-Pour M, Bhat M, Bissada N. Clinical evaluation of two potassium nitrate toothpastes for treatment of dental hypersensitivity. Periodontal Case Rep. 1984;6(1):25-30.	Excluded: conference abstract.
92.	Mason S, Burnett GR, Patel N, Patil A, Maclure R. Impact of toothpaste on oral health-related quality of life in people with dentine hypersensitivity. BMC Oral Health. 2019;22;19(1):226. doi: 10.1186/s12903-019-0919-x	Excluded: single arm study with no comparison group.
93.	Mason S, Young S, Qaqish J, Frappin G, Goyal C. Stain control with two modified stannous fluoride/sodium tripolyphosphate toothpastes: a randomised controlled proof of concept study. J Dent. 2019;91S:100009. doi: 10.1016/j.jjodo.2019.100009	Excluded: the outcome is staining, not dentin hypersensitivity.
94.	Maximiano V, Machado AC, Yoshida ML, Pannuti CM, Scaramucci T, Aranha AC. Nd:YAG laser and calcium sodium phosphosilicate prophylaxis paste in the treatment of dentin hypersensitivity: a double-blind randomized clinical study. Clin Oral Investig. 2019;23:3331-8.	Excluded: the study used laser in office.
95.	McFall WT, Morgan WC Jr. Effectiveness of a dentifrice containing formalin and sodium monofluorophosphate on dental hypersensitivity. J Periodontol. 1985;56(5):288-92. doi: 10.1902/jop.1985.56.5.288	Excluded: the compound tested is formalin (not a compound of interest).
96.	Meffert RM, Hoskins SW. Dentrífico de cloruro de estroncio para combetir la hipersensibilidad dental [Strontium chloride dentifrice to control dental hypersensitivity]. Rev Esp Parad. 1968;6(2):99-107. Spanish.	Excluded: not accessed.
97.	Milleman JL, Milleman KR, Clark CE, Mongiello KA, Simonton TC, Proskin HM. NUPRO sensodyne prophylaxis paste with novamin for the treatment of dentin hypersensitivity: a 4-week clinical study. Am J Dent. 2012;25(5):262-8.	Excluded: in-office treatment.
98.	Miller S, Gaffar A, Sullivan R, Heu R, Truong T, Stranick M. Evaluation of a new dentifrice for the treatment of sensitive teeth. J Clin Dent. 1994;5:71-9.	Excluded: in vitro study.
99.	Mockdeci H, Polonini H, Martins I, Granato AP, Raposo N, Chaves MG. Evaluation of ex vivo effectiveness of commercial desensitizing dentifrices. J Clin Exp Dent. 2017;9(4):e503-e510. doi: 10.4317/jced.53040	Excluded: not in humans.
100.	Monterubbianesi R, Sparabombe S, Tosco V, Profili F, Mascitti M, Hosein A, et al. Can desensitizing toothpastes also have an effect on gingival inflammation? a double-blind, three-treatment crossover clinical trial. Int J Environ Res Public Health. 2020;1;17(23):8927. doi: 10.3390/ijerph17238927	Excluded: the outcome is dental plaque, not dentin hypersensitivity.
101.	Murthy KS, Talim ST, Singh I. A comparative evaluation of topical application and iontophoresis of sodium fluoride for desensitization of hypersensitive dentin. Oral Surg Oral Med Oral Pathol. 1973;36(3):448-58. doi: 10.1016/0030-4220(73)90227-2	Excluded: in-office treatment.
102.	Nabi N, Kashuba B, Lucchesi S, Afflitto J, Furuichi Y, Gaffar A. In vitro and in vivo studies on salifluor/PVM/MA copolymer/NaF combination as an antiplaque agent. J Clin Periodontol. 1996;23(12): 1084-92. doi: 10.1111/j.1600-051x.1996.tb01808.x	Excluded: not in humans and there is no comparision group with other toothpaste.
103.	Nathoo S, Mateo LR, Delgado E, Zhang YP, DeVizio W. Extrinsic stain removal efficacy of a new dentifrice containing 0.3% triclosan, 2.0% PVM/MA copolymer, 0.243% NaF and specially-designed silica for sensitivity relief and whitening benefits as compared to a dentifrice containing 0.3% triclosan, 2% PVM/MA copolymer, 0.243% NaF and to a negative control dentifrice containing 0.243% NaF: a 6-week study. Am J Dent. 2011;24 Spec No A:28A-31A.	Excluded: use of toothpaste for bleaching.

104.	Neuhaus KW, Milleman JL, Milleman KR, Mongiello KA, Simonton TC, Clark C, et al. Effectiveness of a calcium sodium phosphosilicate-containing prophylaxis paste in reducing dentine hypersensitivity immediately and 4 weeks after a single application: a double-blind randomized controlled trial. J Clin Periodontol. 2013;40(4):349-57. doi: 10.1111/jcpe.12057	Excluded: there is no comparison group with other toothpaste.
105.	NYU study identifies new approach for treating tooth hypersensitivity. N Y State Dent J. 2010;76(6):47.	Letter to the editor.
106.	Orbak R, Canakci V, Tezel A. Clinical evaluation of an electron-ionizing toothbrush with a toothpaste containing stannous fluoride in treatment of dentine hypersensitivity following periodontal surgery. Dent Mater J. 2001;20(2):164-71. doi: 10.4012/dmj.20.164	The comparison group evaluates toothbrushes.
107.	Patsouri A, Mavrogiannea A, Pepelassi E, Gaintantzopoulou M, Kakaboura A. Clinical effectiveness of a desensitizing system on dentin hypersensitivity in periodontitis patients. Am J Dent. 2001;24(2):85-92.	Patients underweny periodontal surgery; in-office treatment.
108.	Pearce NX, Addy M, Newcombe RG. Dentine hypersensitivity: a clinical trial to compare 2 strontium densensitizing toothpastes with a conventional fluoride toothpaste. J Periodontol. 1994;65(2):113-9. doi: 10.1902/jop.1994.65.2.113.	The study lacks randomization.
109.	Pepelassi E, Rahiotis C, Peponi E, Kakaboura A, Vrotsos I. Effectiveness of an in-office arginine-calcium carbonate paste on dentine hypersensitivity in periodontitis patients: a double-blind, randomized controlled trial. J Clin Periodontol. 2015;42(1):37-45. doi: 10.1111/jcpe.12319	In-office treatment.
110.	Perminder K, Shahani DR, Antia FE. A comparative study of the desensitizing effects of dentifrices containing sodium monofluorophosphate and formalin. J Indian Dent Assoc. 1985;57(11):453-8.	The study lacks randomization and a clear diagnosis of dentin hypersensitivity, and tests formalin (not a compound of interest).
111.	Pillon FL, Romani IG, Schmidt ER. Effect of a 3% potassium oxalate topical application on dentinal hypersensitivity after subgingival scaling and root planing. J Periodontol. 2004;75(11):1461-4. doi: 10.1902/jop.2004.75.11.1461	The study lacks a comparison with other toothpastes.
112.	Poulsen S. Available evidence does not support use of oxalates for dentine hypersensitivity. Evid Based Dent. 2011;12(2):47. doi: 10.1038/sj.ebd.6400792	Editorial.
113.	Poulsen S, Errboe M, Hovgaard O, Worthington HW. Potassium nitrate toothpaste for dentine hypersensitivity. Cochrane Database Syst Rev. 2001;(2):CD001476. doi: 10.1002/14651858.CD001476	Systematic review.
114.	Poulsen S, Errboe M, Hovgaard O, Worthington HW. Potassium containing toothpastes for dentine hypersensitivity. Cochrane Database Syst Rev. 2006; (3):CD001476. doi: 10.1002/14651858.CD001476.pub2.	Systematic review.
115.	Rahardjo A, Nasia AA, Adiatman M, Maharani DA. Efficacy of a toothpaste containing 5% potassium nitrate in desensitizing dentin hypersensitivity. Asian J Pharm Clin Res. 2016;9 345-7.	In-office treatment.
116.	Rajesh KS, Hedge S, Kumar A, Shetty DG. Evaluation of the efficacy of a 5% calcium sodium phosphosilicate (Novamin) containing dentifrice for the relief of dentinal hypersensitivity: a clinical study. Indian J Dent Res. 2012;23(3):363-7. doi: 10.4103/0970-9290.102228	The study lacks randomization.
117.	Ravishankar P, Viswanath V, Archana D, Keerthi V, Dhanapal S, Lavanya Priya KP. The effect of three desensitizing agents on dentin hypersensitivity: a randomized, split-mouth clinical trial. Indian J Den Res. 2018;29(1):51-5. doi: 10.4103/ijdr.IJDR_458_17	The study used adhesive.
118.	Rigby J. Dentine hypersensitivity: a comparison of five toothpastes used during a 6-week period. Br Dent J. 1987;163(6):181. doi: 10.1038/sj.bdj.4806233	Editorial.
119.	Salvato AR. Potassium chloride dentrifrice as a desensitizing agent. Br Dent J. 1991;171(5):122. doi: 10.1038/sj.bdj.4807628	Editorial.
120.	Samuel SR, Khatri SG, Acharya S, Patil ST. Evaluation of instant desensitization after a single topical application over 30 days: a randomized trial. Aust Dent J. 2015;60(3):336-42. doi: 10.1111/adj.12341	In-office treatment.
121.	Samuel SR, Khatri SG, Achrya S. Clinical evaluation of self and professionally applied desensitizing agents in relieving dentin hypersensitivity after a single topical application: a randomized controlled trial. J Clin Exp Dent. 2014;6(4):e339-43. doi: 10.4317/jced.51439	In-office treatment.

122.	Sanchis Lopez J, Sanchez Gonzalez J, Sanchis Lopes C. Estudio comparativo de dos metodos para el tratamiento de la hipersensibilidad dentinal [Comparative study of 2 methods of treatment of dentin hypersensitivity]. Rev Actual Estomatol Esp. 1988;48(379):47-50. Spanish	The study compares gel to cotton rolls applied on tooth surfaces.
123.	Schafer F, Sun JN, Naeeni MA, Li X. Immediate and sustained reduction of dentine hypersensitivity from hydroxapatite toothpaste. In: Proceedings of the 6th General Session of the Pan European Region of the IADR 2012. Abstract 84.	Abstract of a meeting.
124.	Schlee M, Rathe F, Bommer C, Bröseler F, Kind L. Self-assembling peptide matrix for treatment of dentin hypersensitivity: a randomized controlled clinical trial. J Periodontol. 2018;89(6):653-60.doi: 10.1002/JPER.17-0429	The study used gel.
125.	Schiff T, Delgado E, Zhang YP, Cummins D, DeVizio W, Mateo LR. Clinical evaluation of the efficacy of an in-office desensitizing paste containing 8% arginine and calcium carbonate in providing instant and lasting relief of dentin hypersensitivity. Am J Dent. 2009;22 Spec No A:8A-15A.	The study lacks a comparison group with other toothpastes and analyzes other in-office compasion groups.
126.	Schwarz P, Benz C, Sonnabend E. Untersuchung der Wirksamkeit einer 5%igen KNO3-Zahncreme bei Dentinüberempfindlichkeit [Effectiveness of a 5% KNO3 toothpaste on dentin hypersensitivity]. Dtsch Zahnarztl Z. 1987;42(9):822-5. German.	The study lacks a randomized controlled trial.
127.	Seo MS, Park DS, Jeong CM. The effectiveness of potassium oxalate and sodium fluoride on the reduction of dentinal hypersensitivity. J Dent Res. 1991;70.	Abstract of a meeting.
128.	Seong J, Parkinson CP, Davies M, Claydon, NCA, West NX. Randomised clinical trial to evaluate changes in dentine tubule occlusion following 4 weeks use of an occluding toothpaste. Clin Oral Investig. 2018;22(1):225-33. doi: 10.1007/s00784-017-2103-5	The study analyzes a SEM- visualized silicone impression.
129.	Shapiro WB, Kaslick RS, Chasens AI. The effect of a strontium chloride toothpaste on root hypersensitivity in a controlled clinical study. J Periodontol.1970;41:702-3.	The study lacks a diagnosis confirmed by tactile, cold and air stimuli.
130.	Shapiro WB, Kaslick RS, Chasens AI, Weinstein D. Controlled clinical comparison between a strontium chloride and a sodium monofluorophosphate toothpaste in diminishing root hypersensitivity. J Periodontol. 1970;41(9):523-5. doi: 10.1902/jop.1970.41.9.523	The study lacks a diagnosis confirmed by tactile, cold and air stimuli.
131.	Sharif MO, Iram S, Brunton PA. Effectiveness of arginine-containing toothpastes in treating dentine hypersensitivity: a systematic review. J Dent. 2013;41(6):483-92. doi: 10.1016/j.jdent.2013.01.009	Systematic review.
132.	Sharma S, Shetty NJ, Uppoor A. Evaluation of the clinical efficacy of potassium nitrate desensitizing mouthwash and a toothpaste in the treatment of dentinal hypersensitivity. J Clin Exp Dent. 2012;4(1):e28-33. doi: 10.4317/jced.50665	The study uses a mouthwash comparison group.
133.	Shiau HJ. Dentin hypersensitivity. J Evid Based Dent Pract. 2012;12:220-8. doi: 10.1016/S1532-3382(12)70043-X	Review.
134.	Sidi AD, Wilson RF, Ashley FP, Sidi AD, Wilson RF, Ashley FP. Efficacy of a new toothpaste formulation in reducing dentine hypersensitivity. J Dent Res. 1991;70.	Conference abstract.
135.	Silverman G, Gingold J, Clark GE. The effectiveness of potassium nitrate and sodium MFP dentifrices in reducing dentinal hypersensitivity. J Dent Res. 1988;67.	Conference abstract.
136.	Simoes AL, Fonseca BM, Holleben P, Silva TM, Borges AB. Nicolo R. Clinical evaluation of desensitizing agents on dentin hypersensitivity. In: Proceedings of the General Session of the IADR; 2013. Abstract 3113.	Conference abstract; in-office; split-mouth design.
137.	Singh VB, Govila CP, Mathur RM. Efficacy of desensitizing agents in dentifrices for hypersensitive teeth. J Indian Dent Assoc. 1984;56(8):305-9.	The study fails to report on clinical examination by tactile, cold and air stimuli and to define the time of trial.
138.	Smith BA, Ash MM. A study of a desensitizing dentifrice and cervical hypersensitivity. J Periodontol. 1964;35:222-31.	The study lacks randomization.
139.	Talioti E, Hill R, Gillam DG. The efficacy of selected desensitizing OTC products: a systematic review. ISRN Dent. 2014;77;2014:865761. doi: 10.1155/2014/865761	Systematic review.

140.	Tavss EA, Fisher SW, Campbell S, Bonta Y, Darcy-Siegel J, Blackwell BL, et al. The scientific rationale and development of an optimized dentifrice for the treatment of dentin hypersensitivity. Am J Dent. 2004;17(1):61-70.	In vitro and in vivo study. The in vivo section is part of a study published elsewere [Schiff, T., et al. (2000). “A randomized clinical trial of the desensitizing efficacy of three dentifrices.” Compend Cont Educ Dent. 2000; 21: 4-10 quis 28.
141.	Tirapelli C, Panzeri H, Lara EH, Soares RG, Peitl O, Zanotto ED. The effect of a novel crystallised bioactive glass-ceramic powder on dentine hypersensitivity: a long-term clinical study. J Oral Rehabil. 2011;38(4):253-62. doi: 10.1111/j.1365-2842.2010.02157.x	The study lacks a comparison group with other toothpastes.
142.	Touyz LZ, Stern J. Hypersensitive dentinal pain attenuation with potassium nitrate. Gen Dent. 1999;47(1):42-5.	The study lacks a comparison group and analyzes only one treatment group.
143.	Trevaux. Hyperesthésie dentinaire. Etude comparative d'une pâte aux amines fluorées et d'une pâte au chlorure de strontium [Dentinal hypersensitivity. Comparative study of amine fluoride paste and strontium chloride paste]. Inf Dent. 1989;71(44):4381-3. French.	The study lacks a trial description and randomization.
144.	Tsai WS, Placa SJ, Panagakos FS. Clinical evaluation of an in-office desensitizing paste containing 8% arginine and calcium carbonate for relief of dentin hypersensitivity prior to dental prophylaxis. Am J Dent. 2012;25(3):165-70.	In-office treatment.
145.	Tzanova S, Ivanova Z, Velinova S. Clinical evaluation of dentinal hypersensitivity treatment with 5% potassium nitrate dentifrice. Folia Med (Plovdiv). 2005;47(2):65-9.	The study lacks randomization.
146.	Uchida A, Wakano Y, Fukuyama O, Katsutani Y, Miki T, Kawai J, et al. [The effect of a 10% strontium chloride dentifrice in treatment of dentin hypersensitivity following periodontal surgery]. Nihon Shishubyo Gakkai Kaishi. 1980;22(3):486-91. Japonese. doi: 10.2329/perio.22.486.	Patients underwent periodontal surgery.
147.	Uraz A, Erol-Simsek O, Pehlivan S, Suludere Z, Bal B. The efficacy of 8% Arginine-CaCO3 applications on dentine hypersensitivity following periodontal therapy: a clinical and scanning electron microscopic study. Med Oral Patol Oral Cir Bucal. 2013;18(2):e298-305. doi: 10.4317/medoral.17990	The study compares gel.
148.	Vilhena FV, Polassi MR, Paloco EA, Alonso RC, Guiraldo RD, D'Alpino PH. Effectiveness of toothpaste containing REFIX technology against dentin hypersensitivity: a randomized clinical study. J Contemp Dent Pract. 2020;1;21(6):609-14.	Single-arm study lacking comparison groups.
149.	Wang L, Magalhaes AC, Francisconi-Dos-Rios LF et al. Treatment of dentin hypersensitivity using nano-hydroxyapatite pastes: a randomized three-month clinical trial. Oper Dent. 2016;41(4):e93-e101. doi: 10.2341/15-145-C.	In-office treatment.
150.	Wei SH, Lainson PA, Henderson W, Wolfson SH. Evaluation of dentifrices for the relief of hypersensitive tooth surfaces. Quintessence Int Dent Dig. 1980;11(1):67-73.	Unclear clinical examination; subjective evaluation reports the outcome of the examination (improvement or lack thereof).
151.	West N, Addy M, Jackson RJ, Ridge BD. Comparison of strontium acetate and potassium nitrate dentifrices on dentine hypersensitivity. J Dent Res. 1996;75:1150, Abstract: 1161.	Conference abstract.
152.	West NX. Dentine hypersensitivity: clinical and laboratory studies of toothpastes, their ingredients and acids. Ann Arbor, Wales, Cardiff Medicine; 1995; U083650: 1.	Abstract of a thesis.
153.	West NX, Hooper SM, O'Sullivan D, Hughes N, North M, Macdonald EL, et al. In situ randomised trial investigating abrasive effects of two desensitising toothpastes on dentine with acidic challenge prior to brushing. J Dent. 2012;40(1):77-85. doi: 10.1016/j.jdent.2011.10.010	In situ study.
154.	West NX, Seong J, Davies M. Management of dentine hypersensitivity: efficacy of professionally and self-administered agents. J Clin Periodontol. 2015;42:S256-302. doi: 10.1111/jcpe.12336	Systematic review.
155.	White DJ, Sturzenberger OP, Anastasia JV, Bollmer BW. Intraoral effects of tartar control dentifrices I. Dentin hypersensitivity. J Dent Res. 1992;7:628. Abstract 901.	Conference abstract.
156.	Yan B, Yi J, Li Y, Chen Y, Shi Z. Arginine-containing toothpastes for dentin hypersensitivity: systematic review and meta-analysis. Quintessence Int. 2013;44(9):709-23. doi: 10.3290/j.qi.a30177	Systematic review.
157.	Yang S, Ying K, Wang F, Chen K, Zhou L, Yang Q. [Desensitizing effect of stannous fluoride-containing toothpaste for patients with dentine hypersensitivity: a meta-analysis]. West China J Stomatol. 2016;34(2):150-5. Chinese. doi: 10.7518/hxkq.2016.02.009	Systematic review.

158.	Yang ZY, Li YH, Duan YX, Zhou Z, Zhou H. NovaMin-containing bioactive toothpaste for dentine hypersensitivity: a meta-analysis. Chin J Ev-Based Med. 2014;14:1126-30.	Systematic review.
159.	Yang ZY, Wang F, Lu K, Li YH, Zhou Z. Arginine-containing desensitizing toothpaste for the treatment of dentin hypersensitivity: a meta-analysis. Clin Cosmet Investig Dent. 2016;8:1-14. doi: 10.2147/CCIDE.S95660	Systematic review.
160.	Zhang X, Stomatology DO, Hospital Beijing L. A Comparative study on several common desensitizers in treatment of dentin hypersensitivity. J Clin Exp Med. 2017.	Unaccessed.
161.	Zhu M, Li J, Chen B et al. The effect of calcium sodium phosphosilicate on dentin hypersensitivity: a systematic review and meta-analysis. PloS One. 2015;10(11):e0140176. doi: 10.1371/journal.pone.0140176	Systematic review.
162.	Zinner DD, Duany LF, Lutz HJ. A new desensitizing dentifrice: preliminary report. J Am Dent Assoc. 1977;95(5):982-5. doi: 10.14219/jada.archive.1977.0182	Outcome diagnosis lacks clarity, i.e., not by tactile, cold or air stimuli.
163.	Chad J Anderson, investigator; Procter and Gamble, sponsor. A randomized clinical study of the safety and effectiveness of two dentinal hypersensitivity treatments used with normal oral hygiene. ClinicalTrials.gov [Internet]. Bethesda (United States): U.S. National Library of Medicine; 2014 [cited 2021 Dec 15]. Available from: https://clinicaltrials.gov/ct2/show/NCT02221349	Treatment involves professionally-applied gel.
164.	Johnson & Johnson Consumer and Personal Products Worldwide. Evaluation of the efficacy of two potassium oxalate containing mouthrinses for relieving dentinal hypersensitivity. ClinicalTrials.gov [Internet]. Bethesda (United States): U.S. National Library of Medicine; 2010 [cited 2021 Dec 15]. Available from: https://clinicaltrials.gov/ct2/show/NCT01133379.	Mouthrinse treatment.
165.	GlaxoSmithKline, investigator and sponsor. A clinical study investigating the efficacy of two experimental oral rinses in providing long term relief from dentinal hypersensitivity. ClinicalTrials.gov [Internet]. Bethesda (United States): U.S. National Library of Medicine; 2015 [cited 2021 Dec 15]. Available from: https://clinicaltrials.gov/ct2/show/NCT02542943.	Mouthrinse treatment.
166.	GlaxoSmithKline, investigator and sponsor. A clinical study investigating the efficacy of two experimental oral rinses in providing long term relief from dentinal hypersensitivity. ClinicalTrials.gov [Internet]. Bethesda (United States): U.S. National Library of Medicine; 2015 [cited 2021 Dec 15]. Available from: https://clinicaltrials.gov/ct2/show/NCT02753075	Protocol is mouthwashing and brushing with a fluoride toothpaste.
167.	Michael Lynch, investigator; Johnson & Johnson Consumer and Personal Products Worldwide, sponsor. Assessment of a potassium oxalate containing formulation for the relief of dentinal hypersensitivity. ClinicalTrials.gov [Internet]. Bethesda (United States): U.S. National Library of Medicine; 2014 [cited 2021 Dec 15]. Available from: https://clinicaltrials.gov/ct2/show/NCT02113579	Mouthrinse treatment.
168.	Zangrando, Mariana S Ragghianti, investigator; University os São Paulo, sponsor. Comparative clinical evaluation of three different technologies (novamin, refix and refix-k) in reducing dental hypersensitivity in periodontal patients. ClinicalTrials.gov [Internet]. Bethesda (United States): U.S. National Library of Medicine; 2020 [cited 2021 Dec 15]. Available from: https://clinicaltrials.gov/ct2/show/NCT04422184	Patients showed periodontal disease and received in-office polishing with the assigned dentifrice.
169.	Ebtsam Kassab, investigator; Al-Azhar University, sponsor. The effect of using two desensitizing agents alone and in combination on dentin permeability and hypersensitivity. ClinicalTrials.gov [Internet]. Bethesda (United States): U.S. National Library of Medicine; 2019 [cited 2021 Dec 15]. Available from: https://clinicaltrials.gov/ct2/show/NCT04340869	Unclear treatment.
170.	GSK Clinical Trials, GlaxoSmithKline, investigators and sponsors. A clinical study to evaluate the efficacy of two dentifrices for dentine hypersensitivity. ClinicalTrials.gov [Internet]. Bethesda (United States): U.S. National Library of Medicine; 2015 [cited 2021 Dec 15]. Available from: https://clinicaltrials.gov/ct2/show/NCT02371616	Published as “Young S, Sufi F, Siddiqi M, Maclure R, Holt J. A randomized non-inferiority clinical study to evaluate the efficacy of two dentifrices for dentin hypersensitivity. J Clin Dent. 2016;27(4):97-104”.
171.	GSK Clinical Trials, GlaxoSmithKline, investigators and sponsors. A clinical study investigating the efficacy of an occluding dentifrice in providing relief from dentinal hypersensitivity. ClinicalTrials.gov [Internet]. Bethesda (United States): U.S. National Library of Medicine; 2015 [cited 2021 Dec 15]. Available from: https://clinicaltrials.gov/ct2/show/NCT02751450	Published as “Creeth J, Maclure R, Seong J, Gomez-Pereira P, Budhawant C, Sufi F, Holt J, Chapman N, West N. Three randomized studies of dentine hypersensitivity reduction after short-term SnF2 toothpaste use. J Clin Periodontol. 2019;46(11):1105-15. doi: 10.1111/jcpe.13175”

172.	GSK Clinical Trials, GlaxoSmithKline, investigatora and sponsors. A Clinical study investigating the efficacy of an occluding dentifrice in providing relief from dentinal hypersensitivity. ClinicalTrials.gov [Internet]. Bethesda (United States): U.S. National Library of Medicine; 2017 [cited 2021 Dec 15]. Available from: https://clinicaltrials.gov/ct2/show/NCT02612064	Published as "Creeth J, Maclure R, Seong J, Gomez-Pereira P, Budhawant C, Sufi F, Holt J, Chapman N, West N. Three randomized studies of dentine hypersensitivity reduction after short-term SnF2 toothpaste use. J Clin Periodontol. 2019;46(11):1105-15. doi: 10.1111/jcpe.13175"
173.	GSK Clinical Trials, GlaxoSmithKline, investigators and sponsors. A Clinical study investigating the efficacy of an occluding dentifrice in providing relief from dentinal hypersensitivity ClinicalTrials.gov [Internet]. Bethesda (United States): U.S. National Library of Medicine; 2017 [cited 2021 Dec 15]. Available from https://clinicaltrials.gov/ct2/show/NCT02924350	Published as "Creeth J, Maclure R, Seong J, Gomez-Pereira P, Budhawant C, Sufi F, Holt J, Chapman N, West N. Three randomized studies of dentine hypersensitivity reduction after short-term SnF2 toothpaste use. J Clin Periodontol. 2019;46(11):1105-15. doi: 10.1111/jcpe.13175"
174.	GSK Clinical Trials, GlaxoSmithKline, investigators and sponsors. A clinical study investigating the efficacy of an occluding dentifrice in providing relief from dentinal hypersensitivity ClinicalTrials.gov [Internet]. Bethesda (United States): U.S. National Library of Medicine; 2016 [cited 2021 Dec 15]. Available from: https://clinicaltrials.gov/ct2/show/NCT02923895	Published as "Creeth J, Gallob J, Sufi F, Qaqish J, Gomez-Pereira P, Budhawant C, Goyal C. Randomised clinical studies investigating immediate and short-term efficacy of an occluding toothpaste in providing dentine hypersensitivity relief. BMC Oral Health. 2019; 4;19(1):98. doi: 10.1186/s12903-019-0781-x."
175.	GSK Clinical Trials, GlaxoSmithKline, investigators and sponsors. A clinical study investigating the efficacy of an occluding dentifrice in providing relief from dentinal hypersensitivity. ClinicalTrials.gov [Internet]. Bethesda (United States): U.S. National Library of Medicine; 2016 [cited 2021 Dec 15]. Available from: https://clinicaltrials.gov/ct2/show/study/NCT02731833	Published as "Creeth J, Gallob J, Sufi F, Qaqish J, Gomez-Pereira P, Budhawant C, Goyal C. Randomised clinical studies investigating immediate and short-term efficacy of an occluding toothpaste in providing dentine hypersensitivity relief. BMC Oral Health. 2019; 4;19(1):98. doi: 10.1186/s12903-019-0781-x."
176.	GSK Clinical Trials, GlaxoSmithKline, investigators and sponsors. Assessment of the efficacy of an experimental occlusion technology dentifrice in dentinal hypersensitivity. ClinicalTrials.gov [Internet]. Bethesda (United States): U.S. National Library of Medicine; 2018 [cited 2021 Dec 15]. Available from: https://clinicaltrials.gov/ct2/show/NCT02861664	Published as "Creeth JE, Burnett GR. Efficacy of an experimental occlusion technology toothpaste in the relief of dentinal hypersensitivity: an 8-week randomised controlled trial. Oral Health Prev Dent. 20217;19(1):195-202. doi: 10.3290/j.ohpd.b1075109".
177.	Augusto Elias, investigator; Colgate Palmolive, sponsor. The efficacy of a toothpaste to reduce sensitivity. ClinicalTrials.gov [Internet]. Bethesda (United States): U.S. National Library of Medicine; 2008 [cited 2021 Dec 15]. Available from: https://clinicaltrials.gov/ct2/show/NCT00763269	Toothpastes lacked desensitizing agents.
178.	Abdulrahman Alshehri, investigator; Ministry of Health, Saudi Arabia,. comparison of clinical efficacy of a miswak extract-containing toothpaste to a dentifrice containing potassium nitrate and to a placebo on dentinal hypersensitivity: a randomized clinical trial. ClinicalTrials.gov [Internet]. Bethesda (United States): U.S. National Library of Medicine; 2020 [cited 2021 Dec 15]. Available from: https://clinicaltrials.gov/ct2/show/NCT04179994	Potentially eligible but lacking study results.
179.	Arellano, Francesca Lo Presti, Universidade del Desareollo, investigators ans sponsors. Effectiveness of the use of desensitizing dentifrices based on nanocrystals of hydroxyapatite / 5% potassium nitrate / 1450ppm sodium monofluorophosphate and potassium nitrate 5% / 1426ppm sodium floride in the treatment of dentine hypersensitivity. Test randomized clinical. ClinicalTrials.gov [Internet]. Bethesda (United States): U.S. National Library of Medicine; 2020 [cited 2021 Dec 15]. Available from: https://clinicaltrials.gov/ct2/show/NCT04156152	Potentially eligible but lacking study results.
180.	GSK Clinical Trials, Shanghai Jiao-tong University, investigators and sponsors. An 8 week, randomised, examiner-blind, controlled clinical study to evaluate the efficacy of a stannous fluoride dentifrice in the relief of dentinal hypersensitivity in a chinese. ClinicalTrials.gov [Internet]. Bethesda (United States): U.S. National Library of Medicine; 2021 [cited 2021 Dec 15]. Available from: https://clinicaltrials.gov/ct2/show/NCT04950465	Potentially eligible but lacking study results.
181.	Polyakova, Maria, investigator; Sechenov University, sponsor. The Effect of toothpastes containing ca-hydroxyapatite, fluoroapatite, and mg-zn-hydroxyapatite on dentin hypersensitivity: a randomized clinical trial. ClinicalTrials.gov [Internet]. Bethesda (United States): U.S. National Library of Medicine; 2021 [cited 2021 Dec 15]. Available from: https://clinicaltrials.gov/ct2/show/NCT04896294	Potentially eligible but lacking study results.
